# Discovery of new molecular entities able to strongly interfere with Hsp90 C-terminal domain

**DOI:** 10.1038/s41598-017-14902-y

**Published:** 2018-01-26

**Authors:** Stefania Terracciano, Alessandra Russo, Maria G. Chini, Maria C. Vaccaro, Marianna Potenza, Antonio Vassallo, Raffaele Riccio, Giuseppe Bifulco, Ines Bruno

**Affiliations:** 1 Department of Pharmacy, University of Salerno, via Giovanni Paolo II, 132, 84084 Fisciano, Italy; 2 Department of Science, University of Basilicata, Viale dell’Ateneo Lucano n.10, 85100 Potenza, Italy

## Abstract

Heat shock protein 90 (Hsp90) is an ATP dependent molecular chaperone deeply involved in the complex network of cellular signaling governing some key functions, such as cell proliferation and survival, invasion and angiogenesis. Over the past years the N-terminal protein domain has been fully investigated as attractive strategy against cancer, but despite the many efforts lavished in the field, none of the N-terminal binders (termed “classical inhibitors”), currently in clinical trials, have yet successfully reached the market, because of the detrimental heat shock response (HSR) that showed to induce; thus, recently, the selective inhibition of Hsp90 C-terminal domain has powerfully emerged as a more promising alternative strategy for anti-cancer therapy, not eliciting this cell rescue cascade. However, the structural complexity of the target protein and, mostly, the lack of a co-crystal structure of C-terminal domain-ligand, essential to drive the identification of new hits, represent the largest hurdles in the development of new selective C-terminal inhibitors. Continuing our investigations on the identification of new anticancer drug candidates, by using an orthogonal screening approach, here we describe two new potent C-terminal inhibitors able to induce cancer cell death and a considerable down-regulation of Hsp90 client oncoproteins, without triggering the undesired heat shock response.

## Introduction

Heat shock proteins (Hsps), Hsp27, Hsp70 and Hsp90 are powerful anti-apoptotic proteins involved in vital mechanisms of cancerous cells, such as proliferation, differentiation, metastasis and invasiveness^1,2^. The amplified expression of Hsps is a common feature in human cancers and is associated with increased tumor growth, metastatic potential of tumor cells and resistance to chemotherapy^3^. As a consequence, the inhibition of Hsps might provide a broad and effective strategy in cancer therapy. Among these molecular chaperones, Hsp90 is a key protein that plays a central role in the folding and maturation of many factors, including important signaling proteins with high relevance to human cancer pathways^4^. Many Hsp90 clients are oncogenes that drive a wide range of malignant transformations in which cells have often become “addicted” to Hsp90’s functions^5–7^. Over the past years, Hsp90 has been deeply investigated, from both industry and academic research institutes, as new potential target for cancer and Hsp90 inhibition has, thus, become an attractive therapeutic concept to develop clinically viable antitumor agents (see http://clinicaltrilas.gov). Despite the many progress made in the discovery and development of Hsp90 inhibitors, and the presence of several N-terminal binders (termed “classical inhibitors”) currently in clinical trials in several tumor types, none of these molecules have yet successfully reached the market^8–12^.

These disappointing results may be associated with the N-terminal modulators’ inherent toxicity (that limits their clinical applicable dosages) and with the strong induction of heat shock response (HSR)^3,13–15^, a well-defined compensatory mechanism leading to an increased expression of heat shock proteins, and responsible for N-domain inhibitors resistance^16,17^. In contrast to these modulators, molecules that interfere with Hsp90 C-terminus have been shown to not produce the deleterious HSR emerging, thus, as a promising alternative and a more effective therapeutic anti-cancer strategy^18–22^. So far, for this less-targeted C-terminal domain only a few inhibitors have been disclosed, including both natural products and their synthetic derivatives^18–22^, that interact with the molecular chaperone at non-overlapping sites (due to their ability to bind Hsp90 in its distinct conformational states)^4,23,24^. Indeed, Hsp90 is a large and conformationally dynamic protein that is known to undergo conformational changes associated with remarkable rearrangements in its structure, and, for this reason, it represents a challenging target for structural analysis. In addition to the structural complexity of this protein, the absence of crystal structures of C-terminal Hsp90-inhibitor complexes, represents the main drawback for progress in the field.

Despite the above-mentioned difficulties and the lack of a convincing grasp regarding the exact structural requirements for C-terminal domain interactions, recently we reported the identification of new potent dihydropyrimidinone based Hsp90 inhibitors that target the C-terminal binding pocket^25–28^.

## Results and Discussion

In order to continue our research program aimed at expanding the number of Hsp90 C- terminal inhibitors, we decided to utilize the surface plasmon resonance (SPR)^25–29^ assay for screening a collection of low molecular weight synthetically accessible compounds, selected in order to explore the chemical space encoded by different scaffolds. In more details, a set of forty-eight commercially available small molecules (Table S1, Supplementary Material), endowed with different structural features (Fig. S1, Supplementary Material) was subjected to SPR screening on recombinant Hsp90α for testing their ability to bind to the immobilized protein.

**Figure 1:**
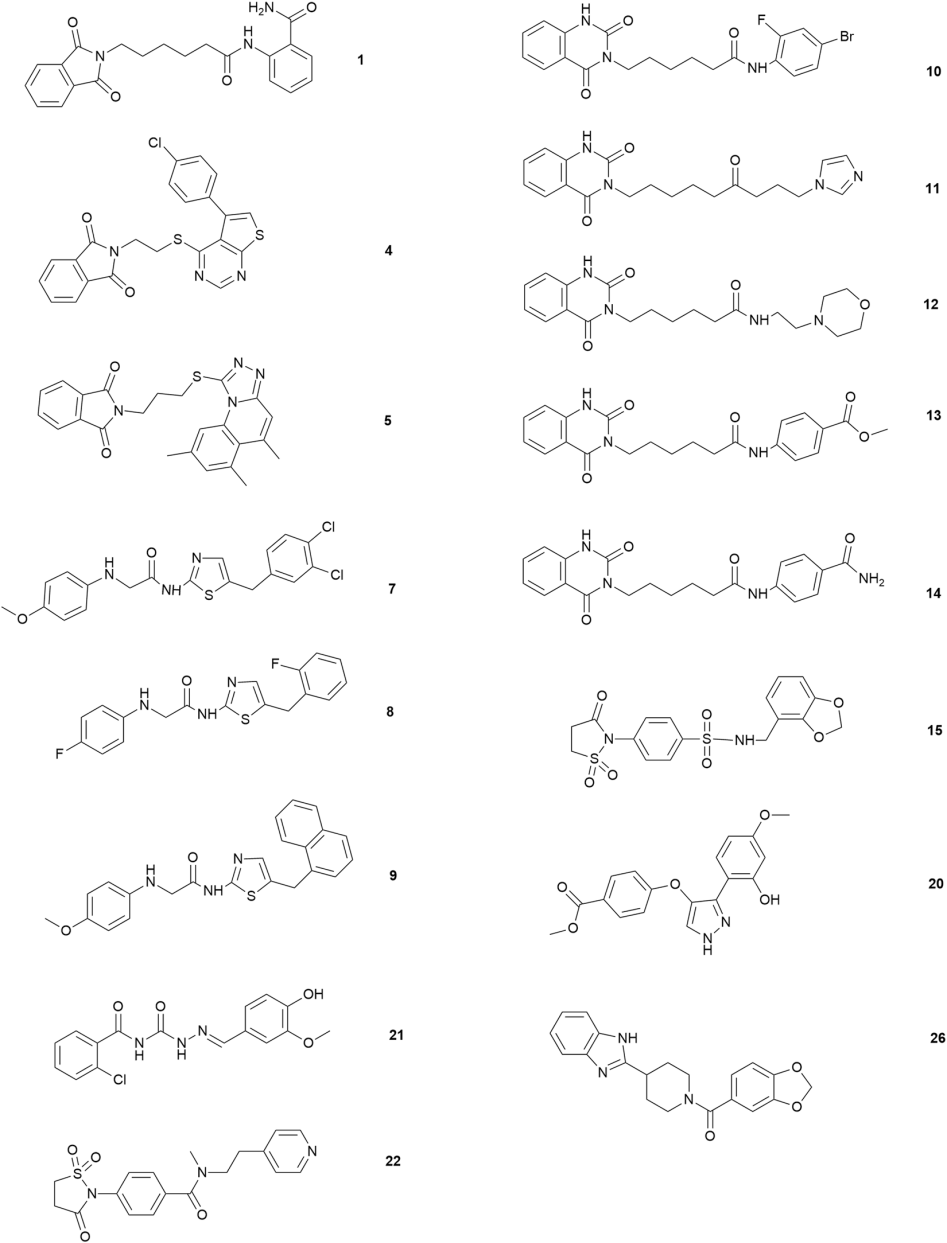
Chemical structures of the selected compounds with high affinity for Hsp90 from SPR screening.

Based on this assay, sixteen compounds with novel chemical scaffolds Fig. 1 have been identified as high affinity leads for the Hsp90 chaperone with low *K*_D_ values (see surface plasmon resonance analyses in material and methods section).

In light of these results, we decided to evaluate their potential anti-proliferative activity against Jurkat (human T-lymphocyte) and U937 (human monocyte form histiocytic lymphoma) cell lines, that were exposed to increasing concentrations of compounds **1**, **4–5**, **7–15**, **20–22**, **26** or novobiocin, a well characterized Hsp90 C-terminal inhibitor^30^. Cells viability was evaluated at 24 or 48 h by MTT assay and IC_50_ values are reported in Tables S2 and S3 (Supplementary Material), respectively. Data showed the best results for **7** and **10** that affected the cell viability in both leukemia-derived cell lines with the lowest IC_50_ values, so they were selected for a deeper biological evaluation. Notably these compounds do not affect the proliferation of non-tumor cell line PHA-stimulated human peripheral blood mononuclear cells (PBMC). In fact, the non-viable cells percentage after 24 or 48 h of treatment with compound **7** (25 µM) (about 6 ± 1.8%, 8 ± 1.5%, respectively), or with with compound **10** (25 µM) (about 7 ± 1.3%, 9 ± 1.8%, respectively), was similar to the value observed in DMSO treated control cells (about 5 ± 0.9%).

Since Jurkat (human leukemic T lymphocyte) cells were about two-fold more susceptible than U937 (human monocytic cell line from histiocytic lymphoma) cells, they were used for additional analysis with the aim of characterizing the mechanism of the observed anti-proliferative effect. In order to discriminate if **7** and **10** affected cell cycle progression and/or inducing cell death, the cells were incubated for 24 or 48 h with a concentration of these two compounds close to their IC_50_ values or two and four times higher. Novobiocin was used at the same conditions with concentrations close to its IC_50_ value (200 µM) or at lower doses (50 or 100 μM).

**Figure 2:**
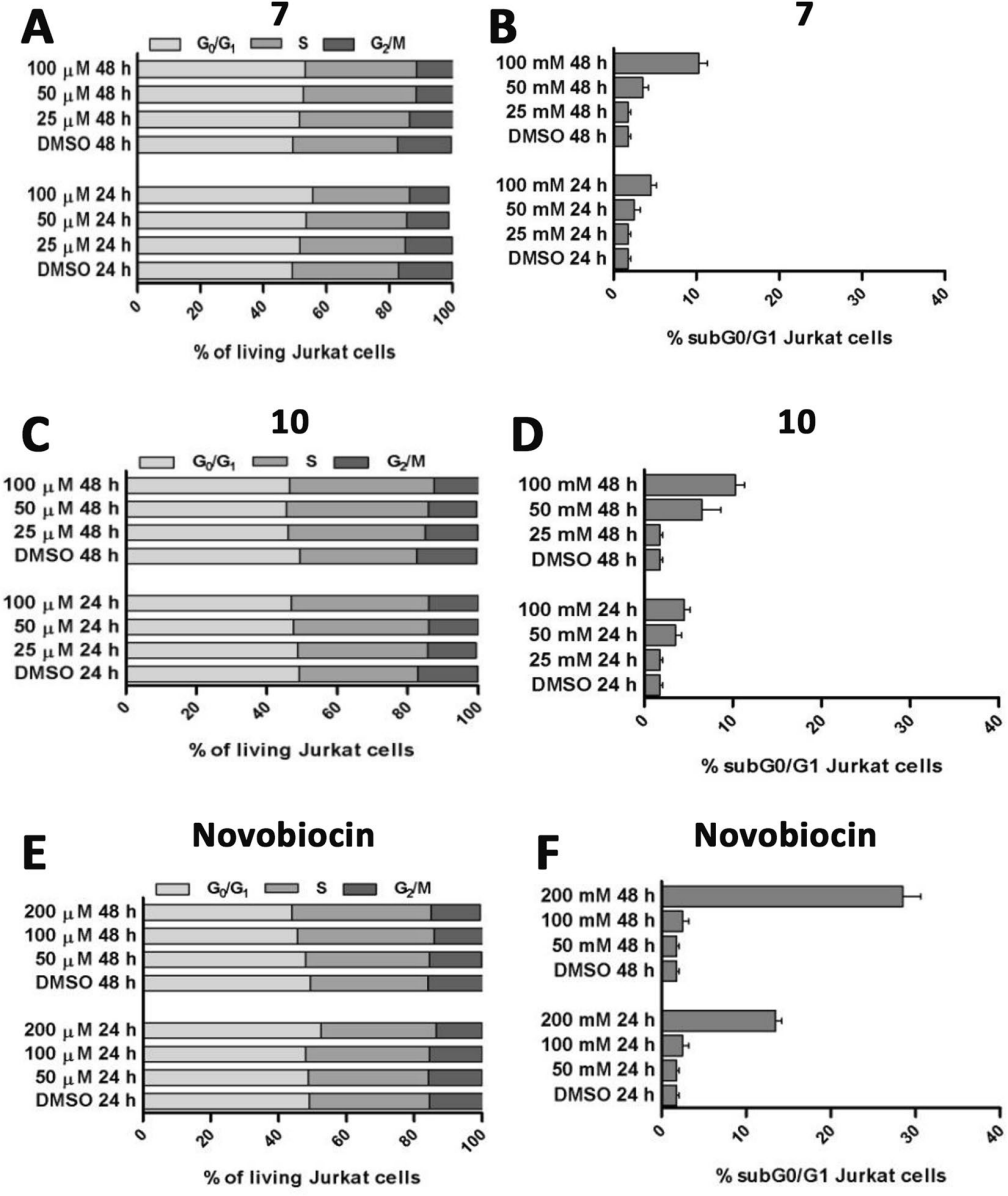
Cell cycle progression analysis of Jurkat cells treated with **7** and **10**. Determination of viable cell cycle distribution was performed in cells treated with compounds **7** (**A**), **10** (**B**) or novobiocin (**C**) at increasing concentrations for 24 and 48 h, and analyzed using flow cytometry PI staining. Results are expressed as means ± SD of three experiment performed in duplicate. The percentage of hypodiploid cells was indicated in (**B**), (**E**) and (**F**).

Cell cycle distribution analysis showed that **7** and **10** moderately prevented the cycle progression by arresting the cells in G_0_/G_1_ or S phase, respectively, after 24 h (Fig. 2A,B) without any significant increase of subG_0_/G_1_ cell fraction, indicative of apoptotic/necrosis cell death (Fig. 2D,E). These dose-dependent cytostatic effects of **7** and **10** were retained even after 48 h (Fig. 2A,B). Conversely, the hypodiploid cells (subG_0_/G_1_ DNA content) increased at 10%, after 48 h cell treatment, with **7** or **10** used at high concentrations (100 µM), as shown in Fig. 2D,E. The novobiocin cell treatment triggered different responses with respect to **7** and **10**, indeed, when it was used at low concentration (50 and 100 µM) for 24 or 48 h, it did not affect cell cycle progression. On the other hand, the exposure to high dose of novobiocin (200 µM), caused cell accumulation in G_0_/G_1_, or S phase (Fig. 2C) with a 14% or 30% of hypodiploid cells after 24 h or 48 h, respectively (Fig. 2F). These data indicate that **7** and **10** induced cytostatic effect after 24 h-exposure and cytostatic/cytotoxic effect after 48 h-exposure on leukemic cells at low doses, compared to novobiocin.

**Figure 3:**
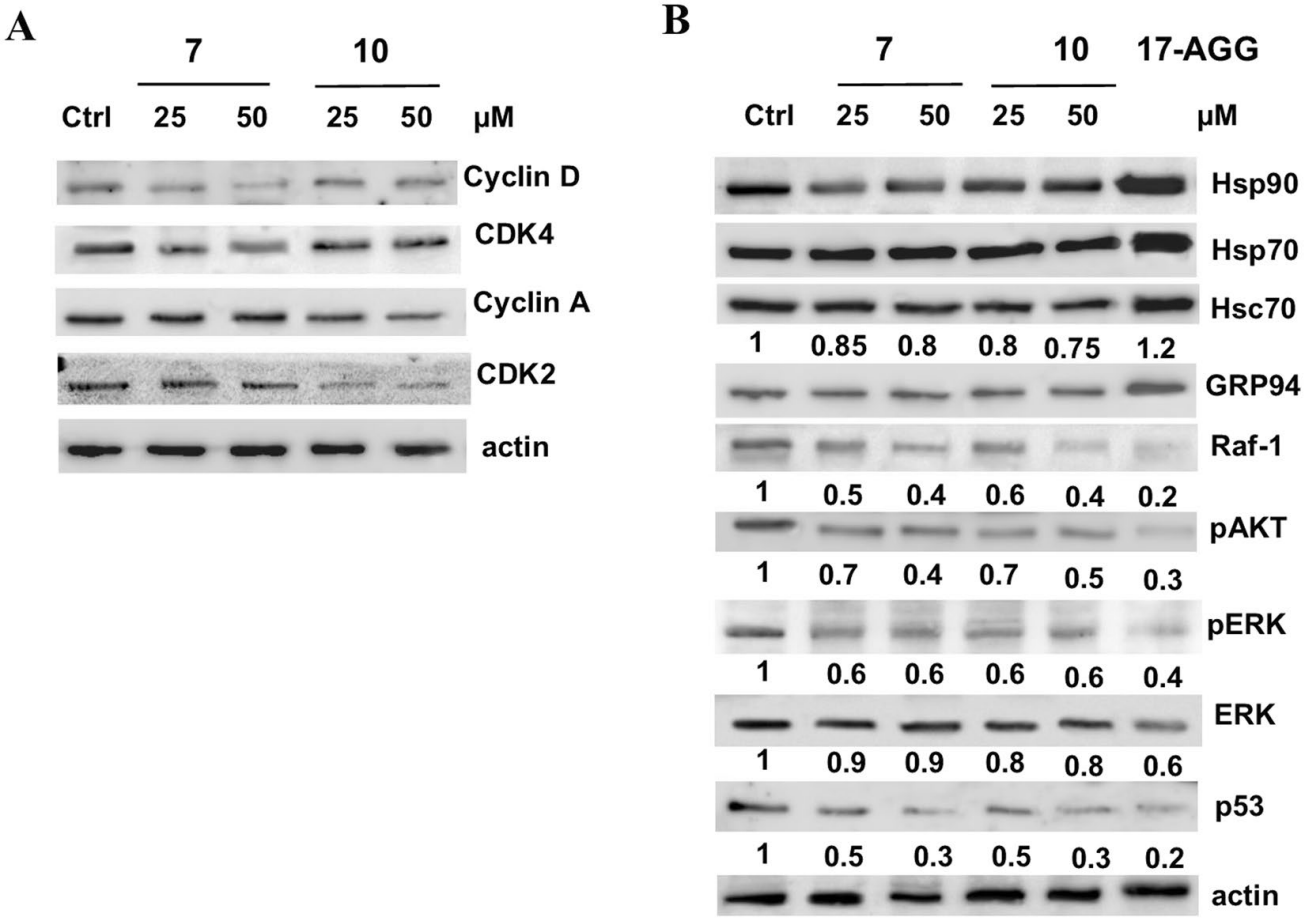
Compounds **7** and **10** affect client protein and cell cycle regulatory protein levels in Jurkat cells. (**A**) The cells were treated with compounds **7** or **10** (25 and 50 µM), or DMSO (control line Ctrl) for 24 h. Equal amounts (40 μg) of total cellular proteins were probed with anti-cyclin A, anti-cyclin D1, anti-CDK2 and anti-CDK4 antibodies. (**B**) The total cellular proteins obtained as in A were probed with specific antibodies. The effects on Hsp90 client-proteins were also analyzed after treatment with 17-AAG (2 µM) for 24 h. Numbers below each panel are densitometric ratio of each band to respective actin levels. The shown blots are representative of three different experiments with similar results.

In addition we analyzed the cell cycle cyclin-dependent kinases, Cyclin A, Cyclin D, CDK2, and CDK4 following the treatment with compounds **7** and **10**. Cyclin D and CDK4 regulates the G0/G1 phase cycle, and their expression levels were slightly down-regulated in Jurkat cells with compound **7** treatment (see Fig. 3A). Similarly compound **10** caused the decrease of Cyclin A and CDK2, involved in normal S-phase progression.

In order to further characterize the most effective compounds and to provide evidence of a *bona fide* inhibition of Hsp90′s activity, the levels of some representative oncogenic Hsp90-client proteins from Jurkat cell lysates, treated with the selected compounds (**7** and **10**), were verified through western blot analysis. As shown in Fig. 3B incubation with **7** and **10** induced a significant degradation (50–60%) of Hsp90-dependent client proteins Raf-1, p-Akt, p-Erk and p53 in a concentration-dependent way, without affecting actin levels, which is not dependent upon Hsp90; a minimal reduction of Hsc70 (15–20%) was observed. Furthermore, the treatment of our compounds did not induce ER chaperone grp94 expression.

Notably, our data demonstrate that these new potent Hsp90 inhibitors did not induce any considerable increase in Hsp90 and Hsp70 protein levels which, conversely is a hallmark resulting from Hsp-90 N-terminal inhibition (Fig. 3). Indeed, 17-AGG, a well-known N-terminal inhibitor^31^, caused a significant up-regulation of Hsp90 and Hsp70 expression levels (Fig. 3).

Thus, basing on these data, we can infer that our compounds have a different modulation effect on Hsp90 than 17-AAG, compatible with Hsp90 C-terminal inhibition. In order to characterize the precise protein region involved in **7** or **10** recognition, a mass spectrometry assisted limited proteolysis strategy was used.

By this approach, through the differences in the proteolytic patterns observed in presence or in absence of putative protein ligands, it is possible to identify the protein regions involved in the molecular interactions^25,32,33^. Limited proteolysis experiments were performed on Hsp90α and on Hsp90α/**7** and on Hsp90α/**10** complexes; moreover, the same experiment was also carried out on the protein interacting with novobiocin, selected as positive control^27,28^.

**Figure 4:**
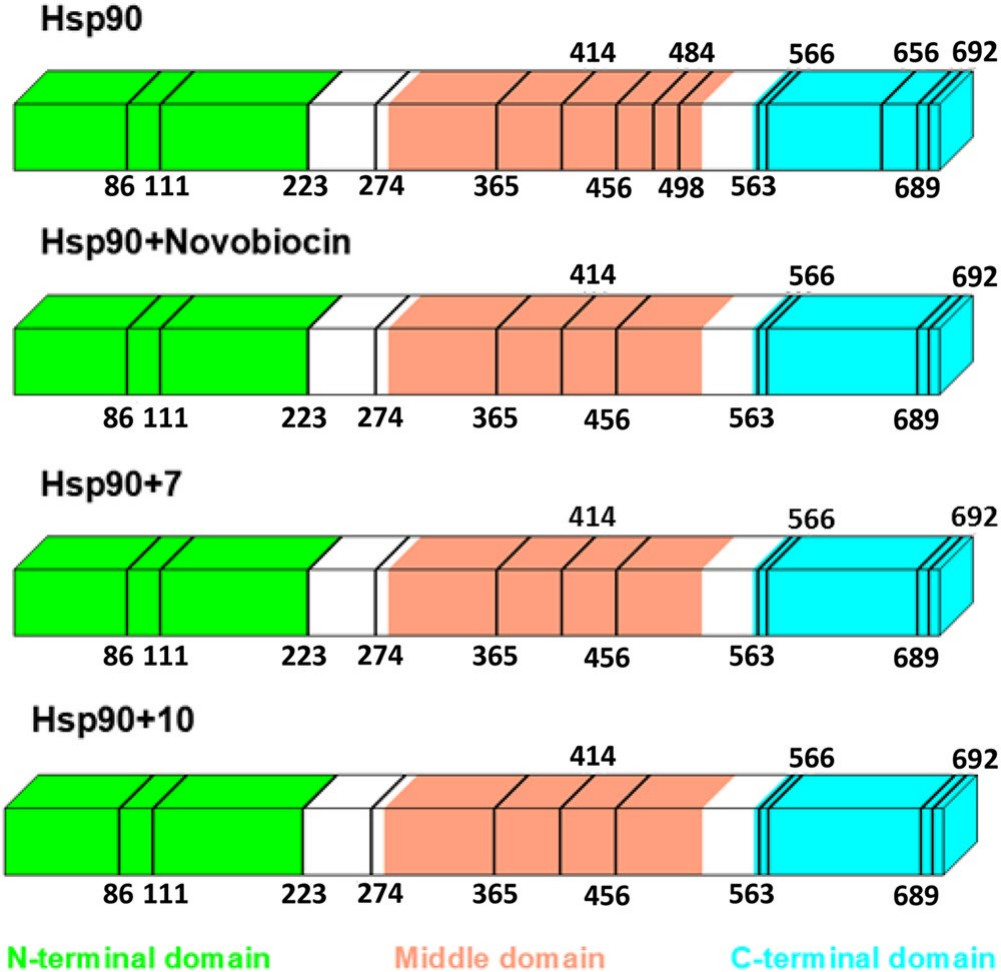
Limited proteolysis experiments. The preferential cleavage sites singled-out in the limited proteolysis experiments performed on recombinant Hsp90α and Hsp90α/Novobiocin, Hsp90α/**7** and Hsp90α/**10** complexes were reported as black lines. The Hsp90α N-terminal, middle and C-terminal domains are represented as light green, pink and light blue bars, respectively.

The preferential trypsin or chymotrypsin cleavage sites, detected for Hsp90α and for the different Hsp90α complexes, identified on the basis of MALDI analysis of the respective digestion mixture, are shown in Fig. 4. A comparison between the results achieved in these experiments suggests that the interaction of Hsp90α with **7** and with **10**, led to a reduced protease accessibility of Lys484, Lys498 and Lys656, thus indicating that the middle and C-terminal domains of Hsp90α are preferentially involved in the molecular binding. In addition, the conformational changes of Hsp90, induced by compound **7** or **10**, is similar to the cleavage sites we detected on Hsp90α/novobiocin^30,34,35^.

**Figure 5:**
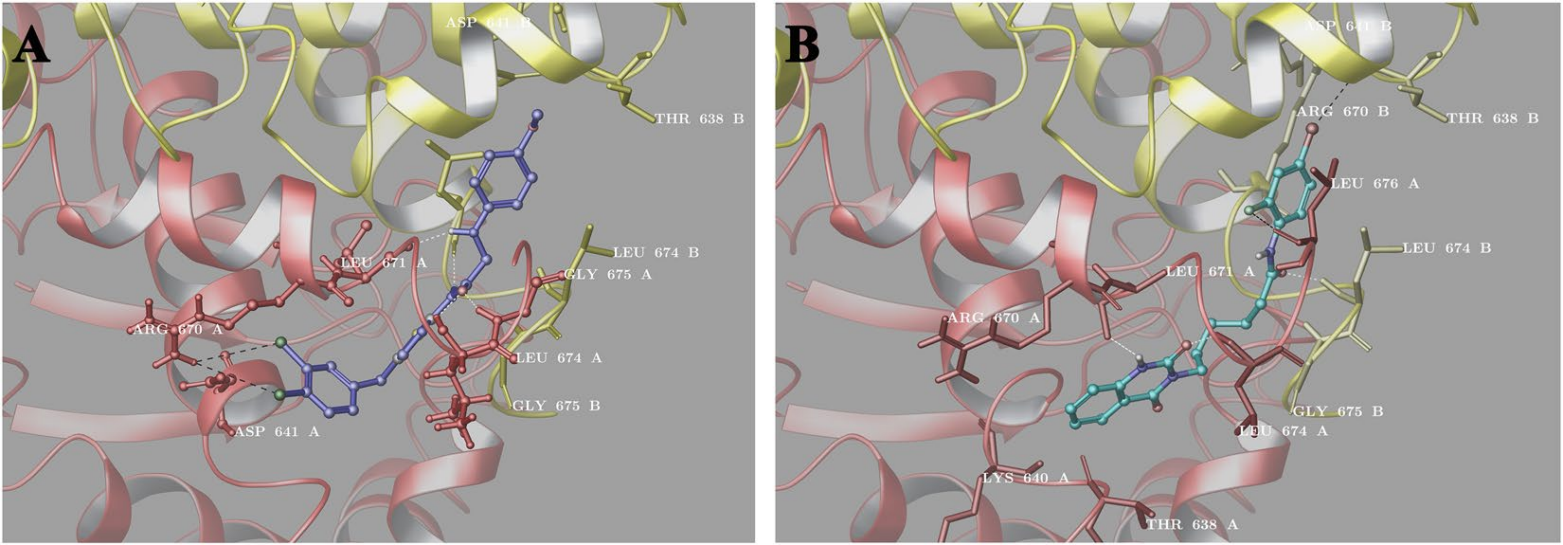
Three dimensional models of **7** (violet sticks, panel A) and **10** (cyan sticks, panel B) with the C-terminal domain of the HSP82 yeast analogue of Hsp90α (chain A is depicted in red and chain B in yellow).

Finally, the molecular basis behind the observed Hsp90 inhibitory activity of the compounds was clarified at molecular level using docking experiments. All the compounds, filtered out by SPR experiments, were docked onto closed active crystal structure of Hsp90α homologue (PDB code: 2CG9)^36^. In more details, we have used the results of limited proteolysis experiments reported above to disclose the domain interacting with our tested compounds, and, during the conformational searches, we have considered a region including the Arg670_Hsp82_ (Lys656_Hsp90_) that is involved in the molecular binding (see above). During our *in silico* analysis, we referred to sequence alignment with the human chaperone protein, reported by Lee *et al*.^37^. In order to assess the binding of tested compounds on Hsp90α, we have performed molecular docking experiments using the induced fit docking protocol (Schrödinger Suite)^38–40^, to account for flexibility of ligands and receptor^41^. In light of the surface plasmon resonance results and the biological data (see Table S3, Supplementary Material), we have disclosed two novel not nature-inspired Hsp90 C-terminal inhibitors (**7** and **10**). For these reasons, during our analysis, we have compared: **7** with its structural related compounds **8** and **9**, and **10** with respect to **11**–**14**. Considering the first group of molecules, from the structural point of view, we have attributed the different biological activity of **7** with respect to **8** and **9** to the diverse substitutions at C-5 of thiazole ring. All the three compounds, in fact, occupy the same region of C-terminal domain, establishing a similar pattern of H-bonds between their phenylamino acetamide and Leu671 and Leu674 of both the chains (A and B, Fig. S2 and S3 Supplementary Material), while the 3,4-dichlorobenzyl moiety of **7** is further involved in a peculiar halogen bond with the side chain of Arg670_ChainA_ (See Fig. 5A). On the other hand, for the second group of molecules (**10**–**14**), we noticed the tendency of these compounds in establishing H-bond contacts between the quinazolinedione moiety and Leu674, Leu676 and Ile672, and between the substituted *N*-hexanamide portion and Asp641_ChainA_ (Fig. S4-S6, Supplementary Material). Furthermore, **10** shows an additional docking pose marked out by the inversion of the *N*-(4-bromo-2-fluorophenyl) hexamide and quinazolinedione moiety in the ligand binding site disclosed above. For this pose, we observed several hydrogen bonds (see Fig. 5B and S5, Supplementary Material) with the receptor counterpart, in detail, between Leu674_ChainB_, Leu671_ChainA_, Gly675_ChainB_ and amide group of *N*-hexanamide and, between the fluorine atom of bromofluorophenyl and Leu676_ChainA_ (see Fig. 5B and S5, Supplementary Material). In addition, its bromide atom forms a halogen bond with the CO of Thr638_ChainB_.

On the other hand_,_ we observed a hydrogen bond between the quinazolinedione and Arg670_ChainA_ and Leu674_ChainA_ (see Fig. 5B and S5, Supplementary Material). For the above considerations, the substitution on *N*-hexanamide is fundamental for biological activity of this type of molecules, thanks to the additional interactions established between this molecular portion and the receptor counterpart (see Table S3 and Fig. S4, Supplementary Material). Considering these two different groups of novel chaperone binders, our structural results suggest that the precise pattern of hydrogen bonds with the leucine aminoacids (Leu671, Leu674, Leu676) direct their placement at the interface of the two Hsp90 A and B chains. Notably, the halogen interactions with Thr638_ChainB_ for **7** and Asp641_ChainA_ for **10** have been confirmed as fundamental interactions suitable for the design of novel more effective C-terminal Hsp90 inhibitors^26–28^.

**Figure 6:**
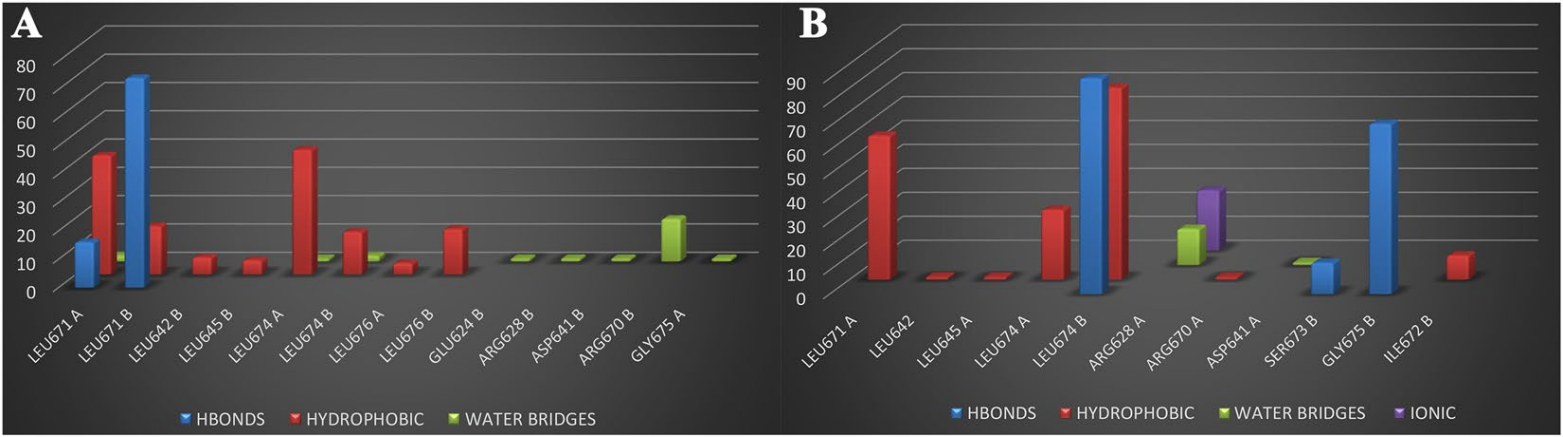
Bar Figure of the protein-ligand (P-L) contacts of **7** (panel A) and **10** (panel B). Hydrogen bonds are represented by blue bars, hydrophobic as red bars, water bridges as green bars, and ionic as purple bars. The stacked bar charts are normalized over the course of the trajectory.

For the considerations above, the best poses of the most active compounds, **7** and **10**, in complex with Hsp90 were used as a starting point for molecular dynamics (MD) simulations (100 ns) in explicit solvent (Desmond software)^42–44^, in order to capture the dynamic nature of protein-ligand interactions and to investigate the ligand affinity and selectivity towards the target^24,45^. In particular, for the analysis of the trajectories we have used the simulation interactions diagram (SID) (Maestro version 10.2)^46^ a tool for exploring protein-ligand interactions. During the simulation, **7** and **10** are located at the interface of the two chains of the C terminal domain, maintaining a good number of interactions (Fig. 6) with the amino acids singled out in the induced fit docking results analysis (see above). From the analysis of the protein-ligand (P-L) contacts (Fig. 6), in addition to further interactions of **7** with Hsp90 (namely with Leu676, Glu624, Asp641) maintained for ≈50% of the simulation time, **7** establishes hydrophobic contact and hydrogen bonds with Leu671 (>80%), with respect to **10**, that mainly interacts with Leu674 (>70%). Hence, the pattern of hydrogen bonds and the number of hydrophobic contacts both with the C-terminal domain (Fig. 6) seem to be the driving forces of the target-ligand complexes.

Therefore, basing on the data here discussed and already reported by us^25,27,28,41^ and by other research groups^45,47–49^, we can only suggest the binding location of our molecules on the protein. This region seems to be not completely overlapped both to the allosteric site of Hsp90 (middle:C-terminal domain)^45,47,48^ and to the second nucleotide pocket on Hsp90-C terminus, for which to date, only computational hypothesis can be proposed because its precise physical location in the CTD as well as its exact physiological role are still unclear^49^.

In conclusion, in the course of our investigations we succeeded to disclose two new structurally unrelated attractive hits with a high binding affinity for C-terminal Hsp90 through a multidisciplinary approach, including SPR protein-ligand interaction measurement, limited proteolysis, molecular docking, molecular dynamics receptor exploration, which allowed us to identify the region of binding of these novel entities. These molecules showed to potently down-regulate the oncogenic clients of the chaperone, the cell cycle cyclin-dependent kinases, inducing cancer-cell death without activating the deleterious HSR response.

These findings are of great interest for progress in the field as they provide an excellent opportunity to expand the chemical space associated with Hsp90 C-terminal inhibition, after a careful molecular optimization process; indeed, the identification of new selective C-terminal modulators are greatly desired because they can represent both attractive anticancer drug candidates and may serve as chemical probes to better clarify the structural requirements needed for an optimal protein-ligand interaction. Such studies are currently underway and will be reported in due course.

## Materials and Methods

### General information

All compounds (**1**–**48**) are available at the stocks of the Otava Chemicals, Otava Ltd. (http://www.otavachemicals.com/) (Otava Codes: are reported in Supplemental Table 1). Chemical structures of **1**–**48** are reported in Supplementary Figure S1.

### Surface Plasmon Resonance Analyses

Recombinant human Hsp90α was purchased from Abcam (Abcam, Cambridge, UK). The Hsp90 inhibitor 17-(Allylamino)-17-demethoxygeldanamycin (17-AAG) was purchased from Sigma-Aldrich. SPR analyses were carried out according to our previously published data^25–28,32,50^. Surface Plasmon Resonance Spectroscopy (SPR) analyses were performed to determine binding of various molecules to full length Hsp90α using a Biacore 3000 optical biosensor equipped with research-grade CM5 sensor chips (GE Healthcare). Hsp90α was coupled to the surface of a CM5 sensor chip using standard amine-coupling protocols, according to the manufacturer’s instructions.

The protein (100 µg mL^−1^ in 10 mM CH_3_COONa, pH 5.0) was immobilized on individual sensor chip surfaces at a flow rate of 5 µL min^−1^ to obtain densities of 8–12 kRU. For the experiments a recombinant Hsp90**α** surface, a BSA surface and one unmodified reference surface were prepared for simultaneous analyses. Compounds **1**–**48**, as well as 17-AAG were dissolved, to obtain 4 mM solutions, in 100% DMSO and diluted 1:200 (v/v) in PBS (10 mM NaH_2_PO_4_, 150 mM NaCl, pH 7.4) to a final DMSO concentration of 0.5%. For each molecule a six-point concentration series were set up, spanning 0 – 0.02 – 0.08 – 0.25 – 1 µM, and for each sample the complete binding study was performed using triplicate aliquots. Changes in mass, due to the binding response, were recorded as resonance units (RU). To obtain the dissociation constant (K_D_) (Table 1), these responses were fit to a 1:1 Langmuir binding model by nonlinear regression using the BiaEvaluation software program provided by GE Healthcare. Simple interactions were suitably fitted to a single-site bimolecular interaction model (A + B = AB), yielding a single K_D_. (Table 1). SPR experiments were performed at 25 °C, using a flow rate of 50 µL min^−1^, with 60 s monitoring of association and 300 s monitoring of dissociation.

**Table 1:** Thermodynamic constants measured by SPR for the interaction between tested compounds and immobilized Hsp90α.

Compound	K_D_ (nM) ± SD	Compound	K_D_ (nM) ± SD
**1**	1.5 ± 0.5	25	No Binding
**2**	No Binding	26	25.7 ± 8.4
**3**	No Binding	27	No Binding
**4**	3.2 ± 1.1	28	No Binding
**5**	14.7 ± 9.4	29	No Binding
**6**	No Binding	30	No Binding
**7**	5.2 ± 3.8	31	No Binding
**8**	2.4 ± 0.6	32	No Binding
**9**	20.9 ± 9.5	33	No Binding
**10**	20.8 ± 8.7	34	No Binding
**11**	79.3 ± 7.2	35	No Binding
**12**	95.1 ± 5.7	36	No Binding
**13**	300 ± 19.3	37	No Binding
**14**	260.2 ± 8.4	38	No Binding
**15**	4.8 ± 2.9	39	No Binding
**16**	No Binding	40	No Binding
**17**	No Binding	41	No Binding
**18**	No Binding	42	No Binding
**19**	No Binding	43	No Binding
**20**	14.2 ± 4.9	44	No Binding
**21**	13.2 ± 2.3	45	No Binding
**22**	4.1 ± 2.6	46	No Binding
**23**	No Binding	47	No Binding
**24**	No Binding	48	No Binding
		17-AAG^a^	388 ± 89

^a^data previously reported^25^.

### Human cell lines and culture conditions

Jurkat (human leukemic T lymphocyte) and U937 (human monocytic cell line from histiocytic lymphoma) cells were obtained from Cell Bank in GMP-IST (Genova, Italy). Cells were maintained in RPMI 1640 medium, containing 10% (v/v) FBS, 2 mM l−glutamine and antibiotics (100 U/mL penicillin, 100 µg/mL streptomycin) purchased from Invitrogen (Carslbad, CA, USA) at 37°C in a 5% CO_2_ atmosphere. Hsp90 inhibitor Novobiocin was purchased from Sigma-Aldrich.

### Anti-proliferation experiments

Jurkat and U937 (2 × 10^4^ /well) cells were seeded in triplicate in 96 well**−**plates and incubated for the 24 h or 48 h in the absence or presence of different concentrations of compounds **1**, **4**–**5**, **7**–**15**, **21**–**22**, **26** (concentration between 10 µM to 100 µM) or Novobiocin (concentration between 50 µM to 500 µM). Stock solutions of compounds (100 mM in DMSO) were diluted just before addition to the sterile culture medium and the final concentration of DMSO was 0.15% (v/v).

The number of viable cells was determined by using a [3–4,5-dimethyldiazol-2-yl]-2,5-diphenyl tetrazolium bromide (MTT, Sigma-Aldrich) conversion assay, according to the method described by Terracciano *et al*. 2016^27,28^. After the indicated treatment, the cells were incubated for additional 3 h at 37 °C, with 25 µL of MTT (5 mg/mL in PBS). The formazan crystals thus formed were dissolved in 100 μl of buffer containing 50% (v/v) *N,N*-dimethylformamide, 20% SDS (pH 4.5). The absorbance was read at 570 nm using an Absorbance Microplate Reader (Titertek multiskan MCC7340, LabSystems, Vienna, VA, USA) equipped with a 620 nm filter. The cell population growth inhibition was also tested by cytometric counting (trypan blu exclusion). IC_50_ values were calculated from cell viability dose**−**response curves and defined as the concentration resulting in 50% inhibition of cell survival, compared to control cells treated with DMSO (see Supplementary Material Tables S2 and S3).

Human peripheral blood mononuclear cells (PBMC), induced to proliferate by phytohemagglutinin (PHA) (10 µg/ml), were used to evaluate cytotoxic effects by trypan blue count of compound **7** and **10**. Human peripheral blood mononuclear cells (PBMC) were isolated from buffy coats of healthy donors (kindly provided by the Blood Center of the Hospital of Battipaglia, Salerno, Italy) by using standard Ficoll−Hypaque gradients. Freshly isolated PBMC contained 92.8 ± 3.1% live cells, were incubated with DMSO or compounds **7** and **10** used at 25 µM (concentrations close to IC_50_ values) for 24 and 48 h.y.

### Cell cycle distribution by flow cytometry

Cell DNA content was measured by propidium iodide (PI) incorporation into permeabilized cells, as described by Nicoletti *et al*. Briefly, the Jurkat cells were treated for 24 h or 48 h with compounds used at the different concentrations. In particular, the compounds **7** or **10** were used at 25 or 50 µM, novobiocin was used at 50, 100 or 200 µM. The treated cells were harvested and incubated with a PI solution (0.1% sodium citrate, 0.1% Triton X-100 and 50 μg/ml of prodium iodide, Sigma-Aldrich, 10 μg/ml Rnase A) for 30 min at room temperature.

The cells were analyzed using FACScalibur flow cytometry (Becton Dickinson, San Josè, CA. The distribution of cells in cell cycle phases was determined using ModFit LT analysis software (Becton Dickinson). The percentage of apoptotic cells is represented as the percentage of hypodiploid cells accumulated at the sub G_0_/G_1_ phase of the cell cycle and was quantified using the CellQuest software (Becton Dickinson). Results were expressed as a mean ± SD of three experiments performed in triplicate.

### Western Blot

The Jurkat cells were seeded in RPMI medium with DMSO or compounds **7** or **10** (25 or 50 µM) or the Hsp90 inhibitor 17-AAG for 24 h. Following the treatment, the cells were trypsinized and homogenized on ice in RIPA lysis buffer (50 mM Hepes, 10 mM EDTA, 150 mM NaCl, 1% NP-40, 0.5% sodium deoxycholate, 0.1% SDS, pH 7.4), supplemented with protease inhibitors cocktail (Sigma-Aldrich). After centrifugation at 4 °C to removed cell debris, an equal protein amount (40 µg) was separated by SDS-PAGE under denatured reducing conditions and was then transferred to nitrocellulose membranes. The blots were blocked with 3% BSA and were incubated at 4 °C overnight with primary antibodies: anti-Hsp 70; anti-Hsp 90α/ß, anti-Raf1, anti-pAkt, anti Erk, anti pErk, anti-p53, anti Hsc70, anti GRP94, anti cyclin A antibodies (Santa Cruz Biotechnology, Inc., Delaware, CA, USA), anti-actin antibody (Sigma-Aldrich) anti CDK2, anti Cyclin D1 and anti CDK4 antibodies (Cell Signaling Technology, Leiden, Netherlands). Appropriate peroxidase-conjugate secondary antibodies were used to detect primary antibodies, and enzymatic signals were visualized by chemiluminescence reagent Pierce ECL (Thermo Scientific, Rockford, IL, USA). Quantitative densitometry analyses were performed using a ImageQuant LAS 4000 system (GE Healthcare Life Sciences,NY, USA).

### Limited Proteolysis

The assays^27,28,50–52^ were conducted on recombinant Hsp90α at 37 °C; PBS 0.1% DMSO, the proteolytic agents trypsin or chymotrypsin, 30 μL of a 3 μM Hsp90α solution for each experiment were used. Hsp90α complexes were obtained by incubating the protein with a 5:1 molar excess of each ligand (**7**, **10** or Novobiocin) at 37 °C for 15 min. Protein and complexes were digested using a 1:100 (w/w) enzyme to substrate ratio. The extent of the proteolysis was monitored on a time-course basis by sampling the reaction mixture at different time intervals (5, 15, and 30 min of digestion). Samples were analyzed by MALDI-TOF/MS instrument using a MALDI micro MX (Waters).

Mass data were processed using the Masslynx software (Waters).

Preferential hydrolysis sites on Hsp90α under different conditions were identified on the basis of the fragments released during enzymatic digestions.

### Statistical analysis

All the reported data represent the mean ± standard deviation (SD) of at least two independent experiments, performed in triplicate. Where necessary, data were statistically compared by Student’s t-test; the statistical significance of DNA content between cells group was examined in the two-way analysis of variance (ANOVA) with Bonferroni post-test analysis using GraphPad Prism 5 software. Differences were considered significant if *p* < 0.05.

### Molecular docking studies

Schrödinger Protein Preparation Wizard workflow^46^ was used to prepare the ATP-bound active state of Hsp82, a yeast Hsp90α homologue (PDB code: 2CG9)^37^ removing the water molecules that were found 5 Å or more and cap termini were included. Additionally, all hydrogen atoms were added and bond orders were assigned. Chemical structures of investigated compounds were built with Maestro’s Build Panel (version 10.2)^46^ and subsequently processed with LigPrep (version 3.4)^53^ in order to generate all the possible tautomers and protonation states at a pH of 7.4 ± 1.0; the resulting ligands were finally minimized employing the OPLS 2005 force field.

### Induced Fit Docking

The Induced Fit Docking protocol is composed of job sequence in which ligands are docked with Glide (first step), then Prime Refinement is used to allow the receptor to relax (second step), and the ligands are redocked into the relaxed receptor with Glide (third step).

Binding site for the first Glide docking phase (Glide Standard Precision Mode) of the Induced Fit Workflow (Induced Fit Docking, protocol 2015–2, Glide version 6.4, Prime version 3.7, Schrödinger)^38–40^ is calculated on the 2CG9 structure^37^, mapping onto a grid with dimensions of 36 Å (outer box) and 20 Å (inner box), centered on residues 628–630, 640–641, 670–675 (Hsp90 residues numbering as in the PDB entry 2CG9). Maestro’s default protocol was used for the first (Initial Glide docking) and the second step (Prime Induced Fit) considering 20 poses per ligand; these poses were retained from the initial docking and then were passed to Prime (Prime version 3.7, Schrödinger 2015), for the Prime refinement step. Finally, the ligands were re-docked (third step) into their corresponding low energy protein structures (Glide Extra Precision Mode) with resulting complexes ranked according to GlideScore.

### Molecular Dynamics Simulations

The starting structures for the Molecular Dynamics (MD) simulations were prepared with the System Builder in Desmond^42,43^. Na^+^ ions were added to system the to ensure electroneutrality, and the SPC^54^ (simple point charge) water model was used for solvation in a rectangular box with a 10 Å buffer distance, resulting in a system with approximately 178000 atoms. OPLS-2005 force field parameters available in the Schrödinger Suite was used for the entire system (protein and substrates). The MD simulation workflow was run with the default parameters in the Maestro interface to Desmond^42,43^, accounting for a total simulation time of 100 ns, using a recording interval of 1.2 ps, and an ensemble class NPT (300 K and 1.01 bar). Before the simulation, a relaxation of the system was performed using the default equilibration protocol in Desmond^42,43^, with multisim procedure, which is a vital step to prepare a molecular system for production-quality MD simulation. In particular, Maestro’s default relaxation protocol was used, which have included two stages of minimization (restrained and unrestrained) followed by four stages of MD runs with gradually diminishing restraints.

## Electronic supplementary material

Supplementary Information
