# Supplementary material for: Discovery of new molecular entities able to strongly interfere with Hsp90 C-terminal domain
Source: Sci Rep. 2018 Jan 26;8:1709. doi: 10.1038/s41598-017-14902-y (PMC5786060; doi:10.1038/s41598-017-14902-y)
Supplement: Supplementary file 1 — Supplementary Information [file 41598_2017_14902_MOESM1_ESM.pdf]

Supplementary Material

Discovery of new molecular entities able to strongly interfere with Hsp90 C-terminal domain

Stefania Terracciano[1], Alessandra Russo[1], Maria G. Chini[1], Maria C. Vaccaro[1], Marianna Potenza[1], Antonio Vassallo[2], Raffaele Riccio[1], Giuseppe Bifulco[1], and Ines Bruno[1,*]

[1]Department of Pharmacy, University of Salerno, via Giovanni Paolo II, 132, 84084, Fisciano, Italy. Fax: +39 089 969602; Tel: +39 089 969741; Tel: +39 089 969743 E-mail: brunoin@unisa.it,

[2]Department of Science, University of Basilicata, Viale dell'Ateneo Lucano n.10, 85100 Potenza, Italy

Supplementary Table S1. Otava Codes of compounds **1-48**.

Compound	Otava Code	Compound	Otava Code
**1**	107620105	**25**	7119920408
**2**	103970196	**26**	1068731
**3**	127460318	**27**	6237462
**4**	7212091019	**28**	129661070
**5**	7020141371	**29**	103490002
**6**	1678691	**30**	1098656
**7**	7715460181	**31**	7020102417
**8**	7715460264	**32**	1089672
**9**	7715460449	**33**	4355978
**10**	1099180	**34**	2194629
**11**	1157839	**35**	4355978
**12**	1157842	**36**	7020721800
**13**	6238190	**37**	1095656
**14**	6238227	**38**	1101808
**15**	7020470041	**39**	7510791436
**16**	7020470326	**40**	1110852
**17**	7020470389	**41**	1101770
**18**	7020530643	**42**	6243033
**19**	7020601381	**43**	7217221018
**20**	125180086	**44**	7010060072
**21**	7113640512	**45**	1157863
**22**	7020532432	**46**	1177455
**23**	6237280	**47**	7010150281
**24**	127442333	**48**	7119461545

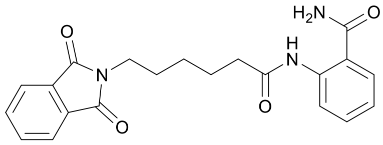

1

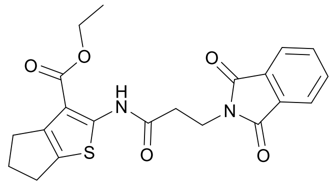

2

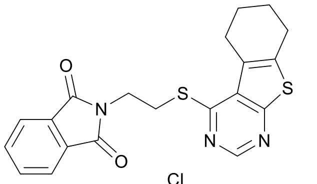

3

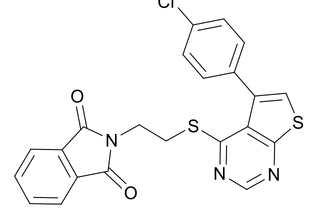

4

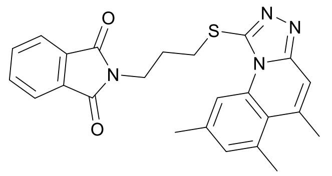

5

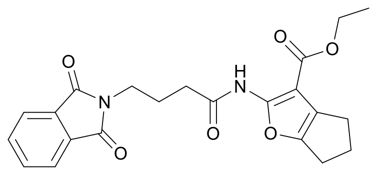

6

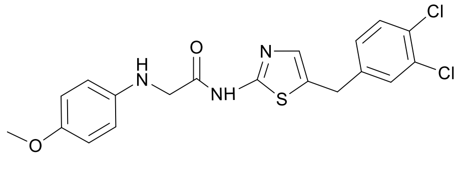

7

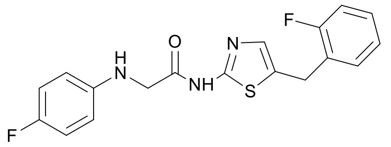

8

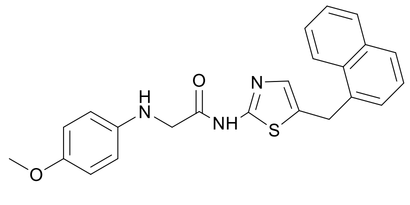

9

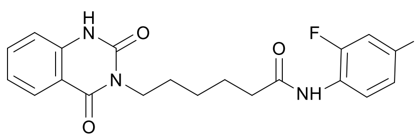

10

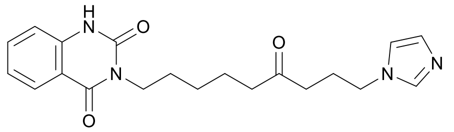

11

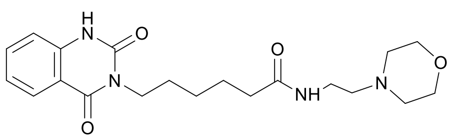

12

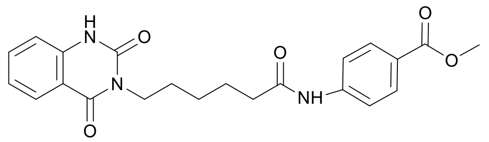

13

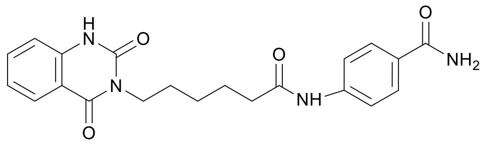

14

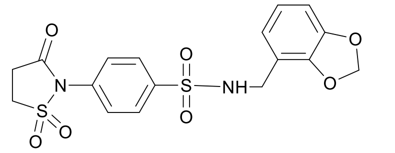

15

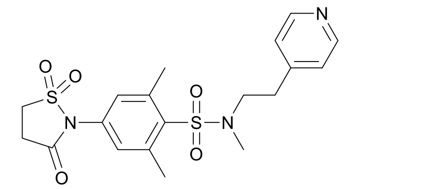

16

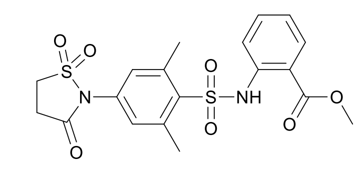

17

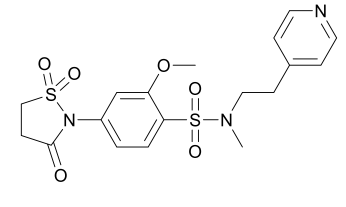

18

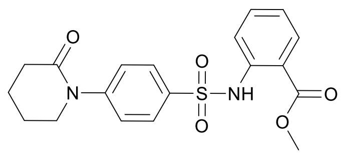

19

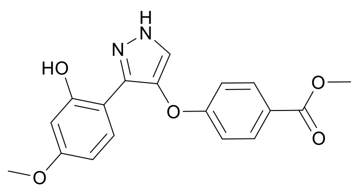

20

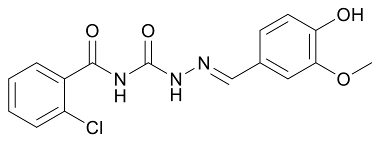

21

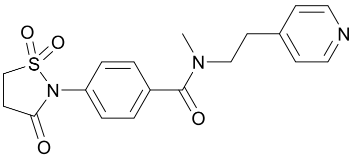

22

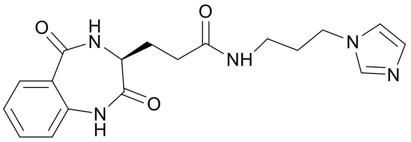

23

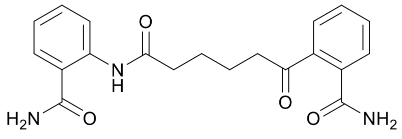

24

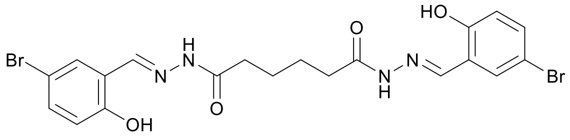

25

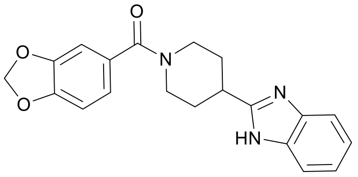

26

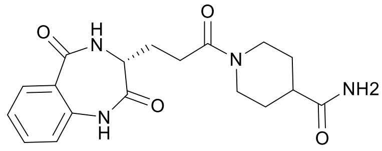

27

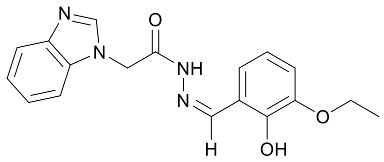

28

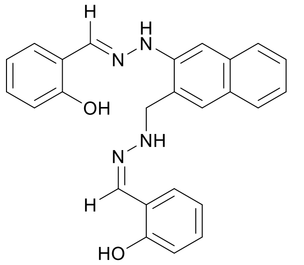

29

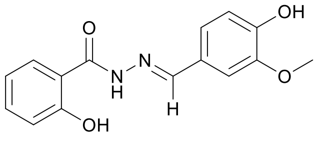

30

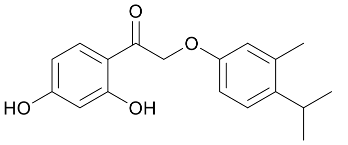

31

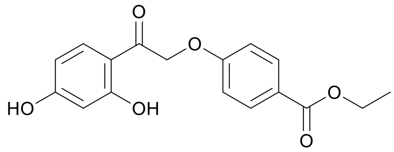

32

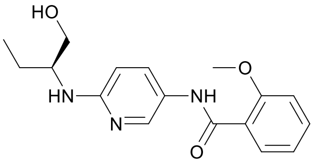

33

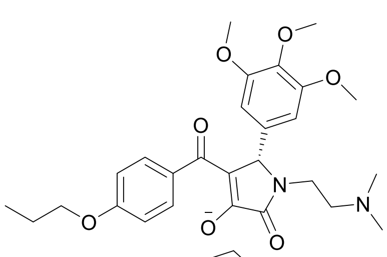

34

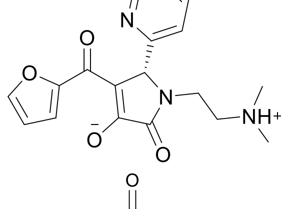

35

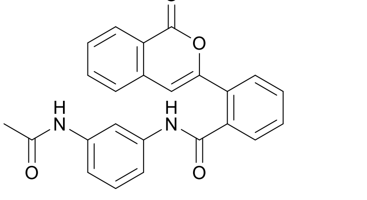

36

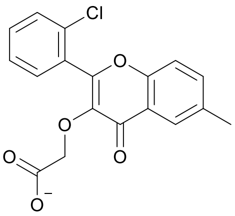

37

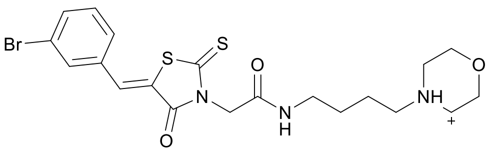

43

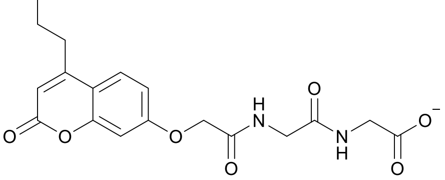

38

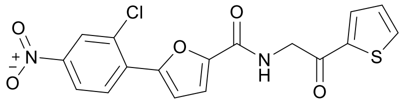

44

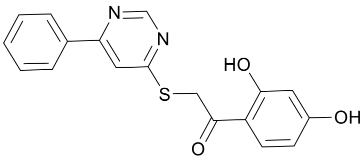

39

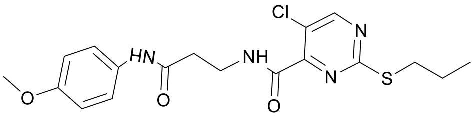

45

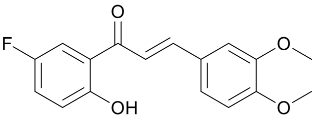

40

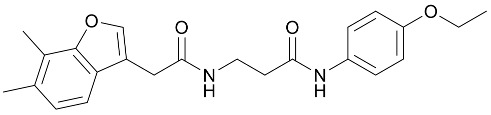

46

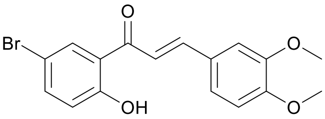

41

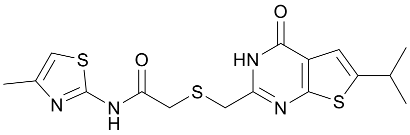

47

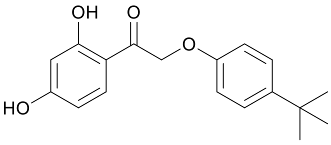

42

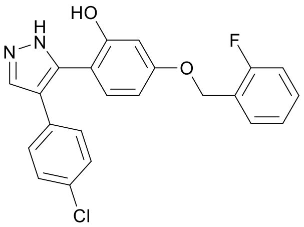

48

Supplementary Figure S1. Chemical structures of compounds **1-48**.

Supplementary Table S2. IC_{50} values of **1**, **4-5**, **7-15**, **21-22**, **26** on human monocytic cell line U937 from histiocytic lymphoma**.**

Compound	IC_{50} (µM) 24h	IC_{50} (µM) 48h
**1**	>100	>100
**4**	>100	>100
**5**	91.2 ± 1.7	91.2 ± 1.2
**7**	**51.2 ± 0.8**	**51.0 ± 0.7**
**8**	not active	not active
**9**	97.3 ± 1.5	95.3 ± 0.9
**10**	**52.0 ± 0.5**	**50.0 ± 0.7**
**11**	90.1 ± 1.2	90.3 ± 1.3
**12**	>100	>100
**13**	98.3 ± 0.9	97.0 ± 1.1
**14**	>100	>100
**15**	not active	not active
**21**	not active	not active
**22**	not active	not active
**26**	>100	>100
**Novobiocin**	232.5± 1.1	190.4± 1.5

Supplementary Table S3. IC_{50} values of **1**, **4-5**, **7-15**, **21-22**, **26** on human leukemic T lymphocyte cell line Jurkat.

Compound	IC_{50} (μM) 24h	IC_{50} (μM) 48h
**1**	>100	>100
**4**	>100	>100
**5**	100.0 ± 1.4	98.3 ± 0.9
**7**	**25.1 ± 0.4**	**22.0 ± 0.8**
**8**	not active	not active
**9**	90.2 ± 0.5	85.5 ± 0.7
**10**	**26.1 ± 0.7**	**23.5 ± 1.2**
**11**	48.2 ± 0.8	42.5 ± 0.5
**12**	>100	>100
**13**	56.8 ± 1.3	54.0 ± 0.5
**14**	>100	>100
**15**	not active	not active
**21**	not active	not active
**22**	not active	not active
**26**	>100	>100
**Novobiocin**	170.6 ± 1.1	150.5 ± 0.7

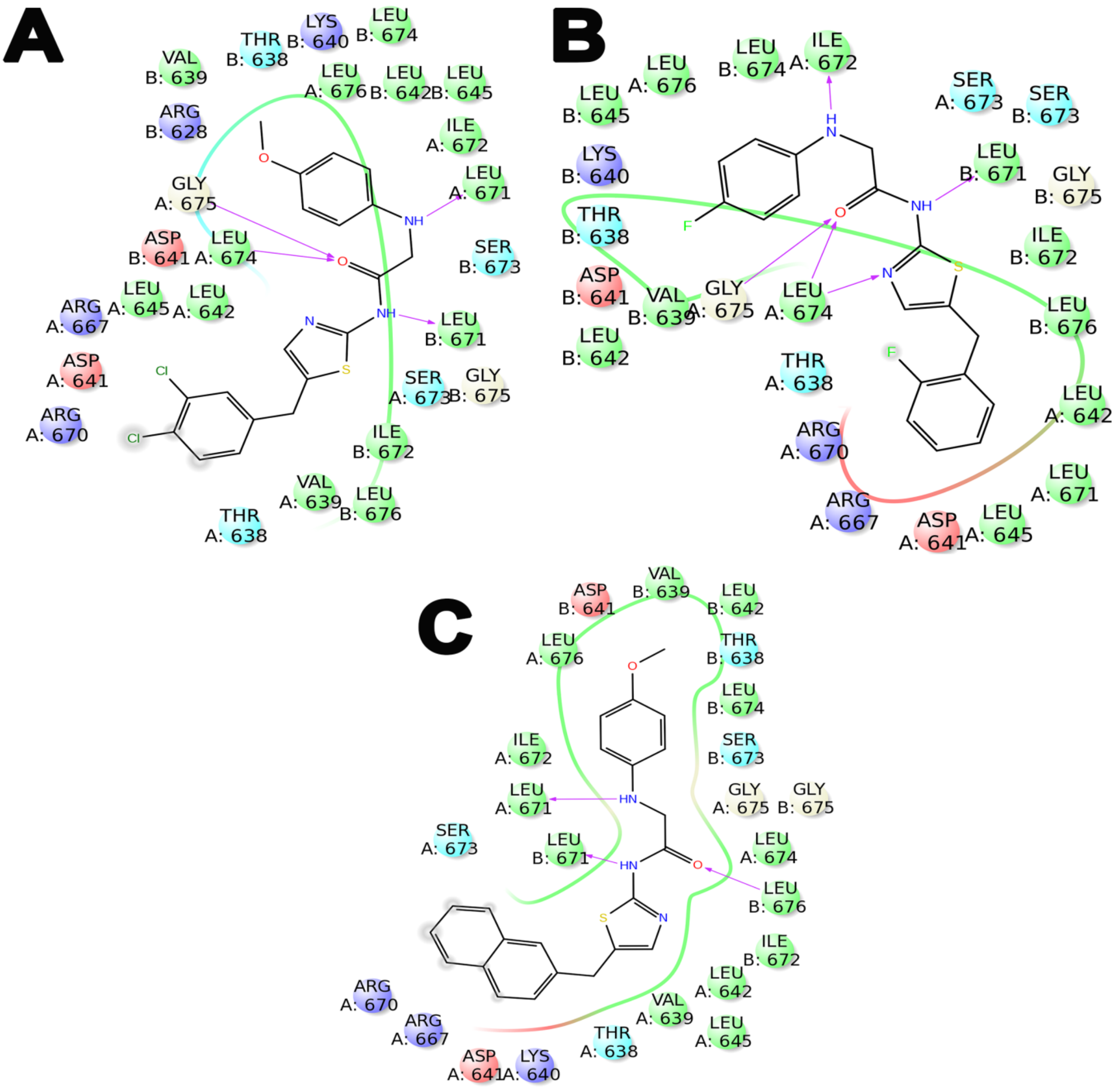

Supplementary Figure S2. 2D diagram interactions (panel A, B and C) of **7**, **8** and **9** with C-terminal domain of HSP82 yeast analogue of Hsp90α (PDB: 2CG9). Positive charged residues are colored in violet, negative charged residues are colored in red, polar residues are colored in light blue, hydrophobic residues are colored in green. H-bond (side chain) are reported as dotted pink arrows.

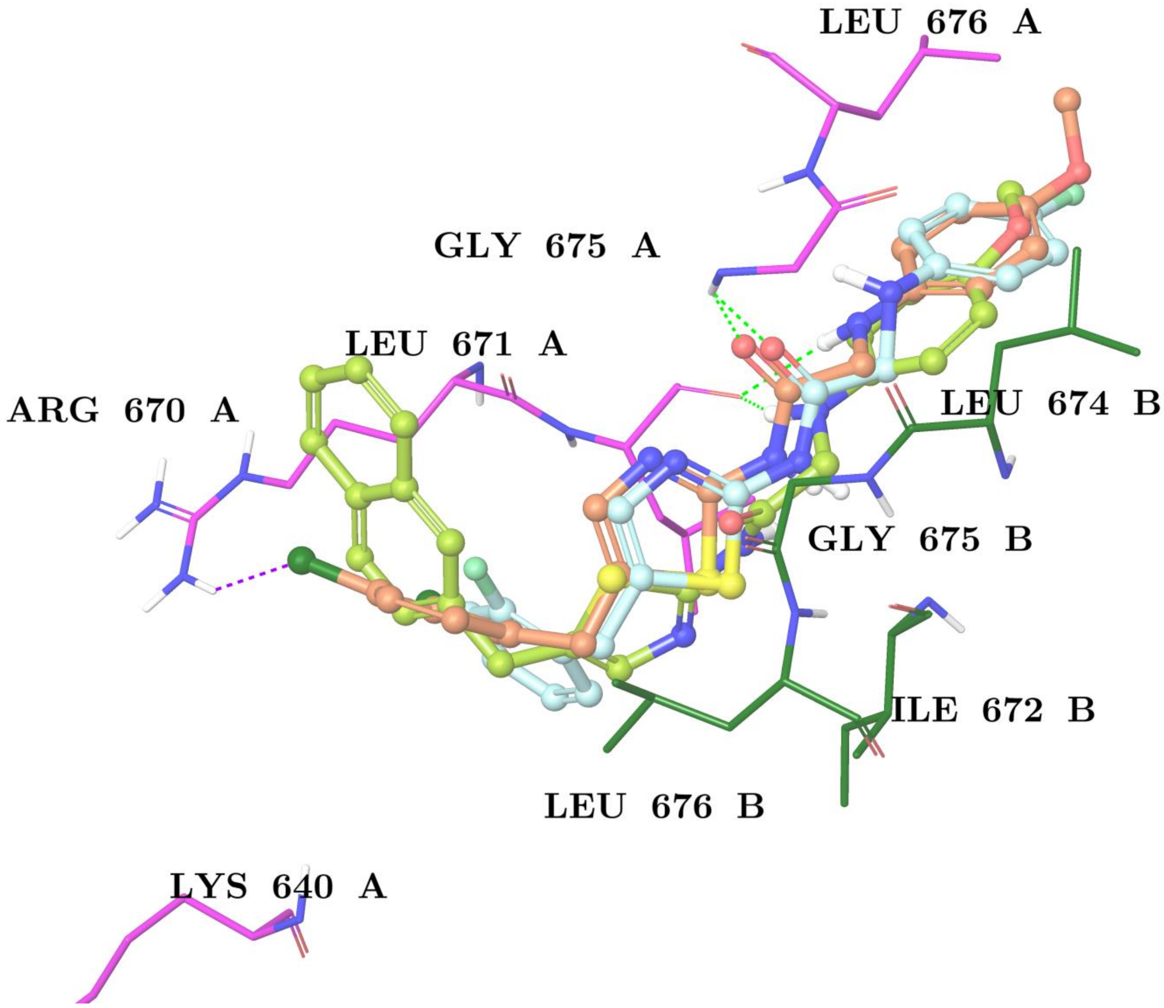

Supplementary Figure S3. Superimposition of **7** (light pink sticks), **8 (**sky-blue sticks) and **9** (light green sticks) with C-terminal domain of HSP82 yeast analogue of Hsp90α (PDB: 2CG9). The protein is depicted by purple (chain A) and green (chain B) sticks.

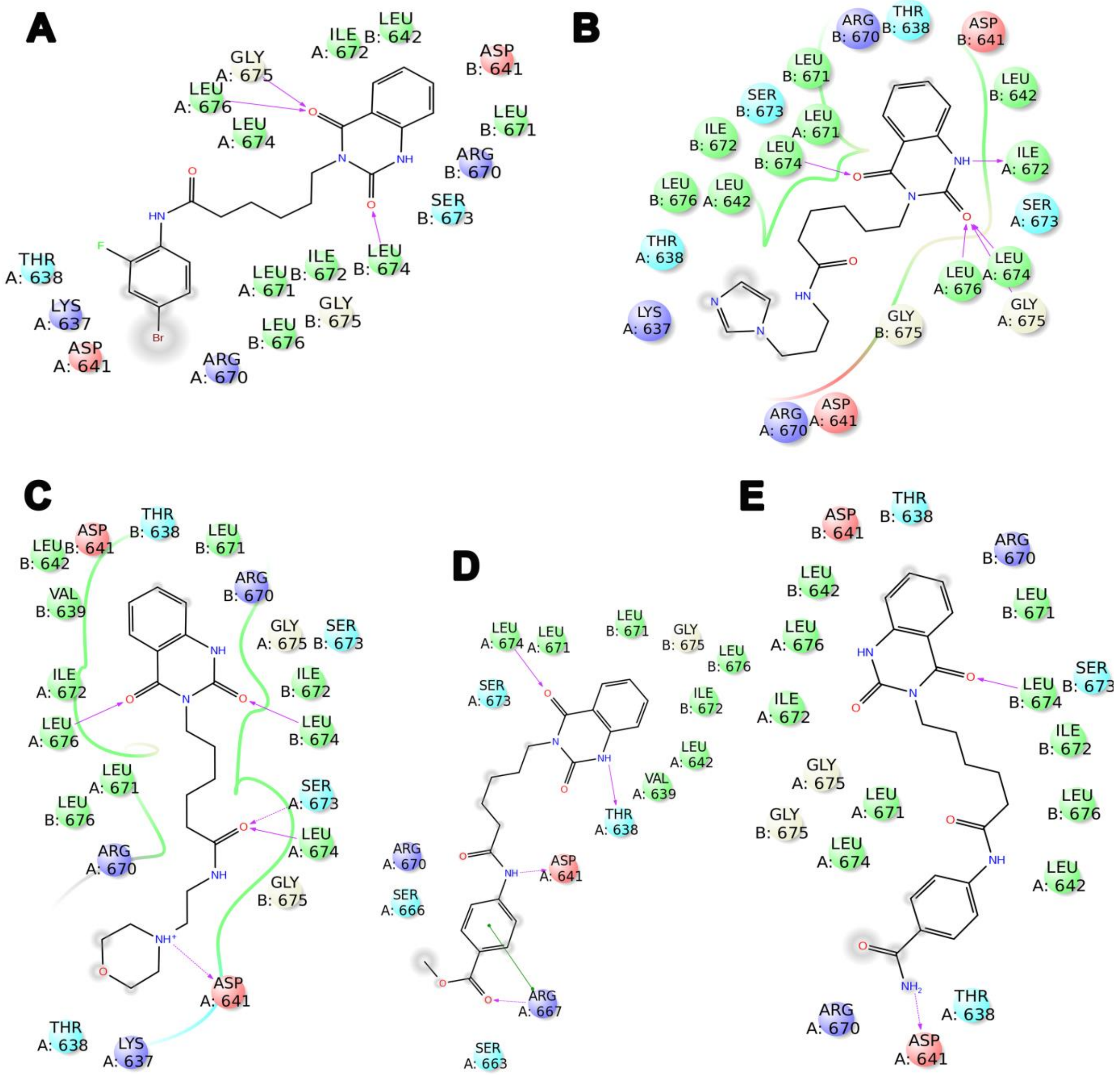

Supplementary Figure S4. 2D diagram interactions (panels A, B, C, D and E) of **10**, **11**, **12**, **13** and **14** with C-terminal domain of HSP82 yeast analogue of Hsp90α (PDB: 2CG9). Positive charged residues are colored in violet, negative charged residues are colored in red, polar residues are colored in light blue, hydrophobic residues are colored in green. The π-π stacking interactions are indicated as green lines, and H-bond (side chain) are reported as dotted pink arrows.

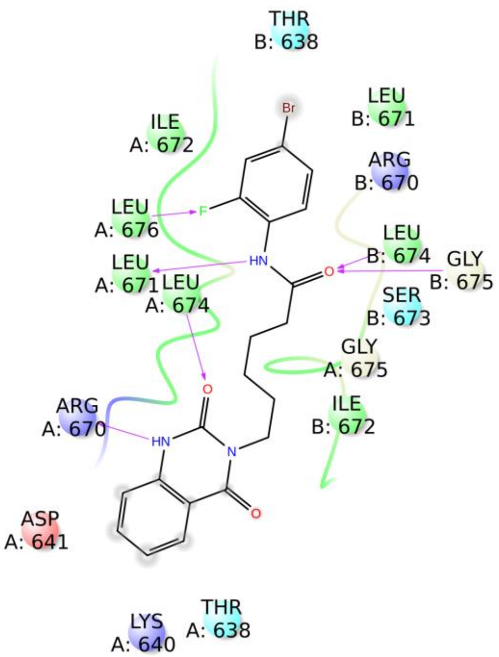

Supplementary Figure S5. 2D diagram interactions of second binding mode of **10** with C-terminal domain of HSP82 yeast analogue of Hsp90α (PDB: 2CG9). The positive charged residues are colored in violet, negative charged residues are colored in red, polar residues are colored in light blue, hydrophobic residues are colored in green. H-bond (side chain) are reported as dotted pink arrows.

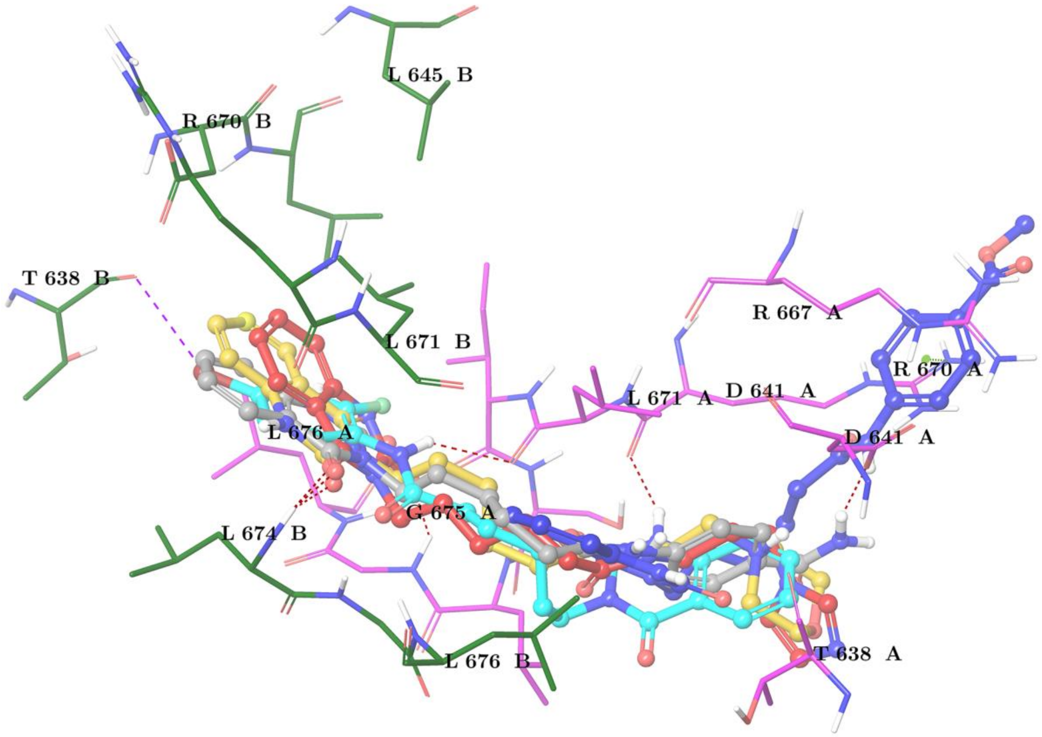

Supplementary Figure S6. Superimposition of **10** (cyan sticks), **11** (red sticks), **12** (yellow sticks), **13** (blue sticks), and **14** (grey sticks) with C-terminal domain of HSP82 yeast analogue of Hsp90α (PDB: 2CG9). The protein is depicted by purple (chain A) and green (chain B) sticks.

1

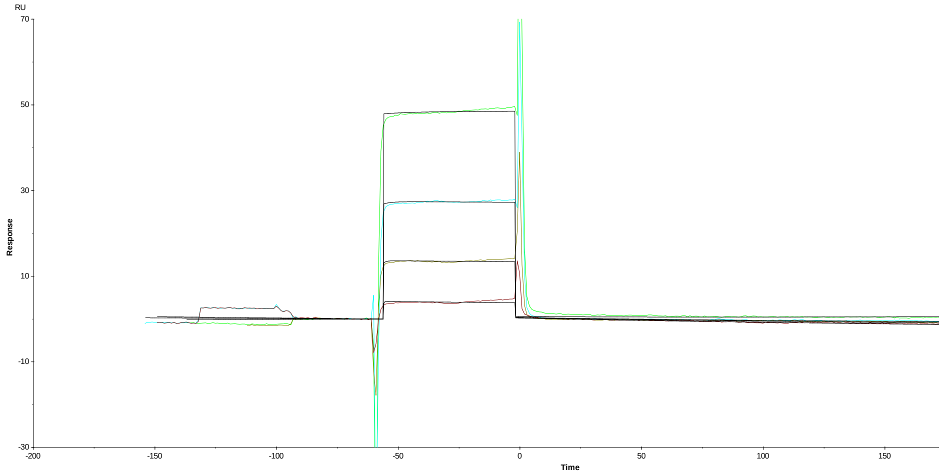
RU
70
50
30
10
-10
-30
-200
-150
-100
-50
0
50
100
150
Response
Time

4

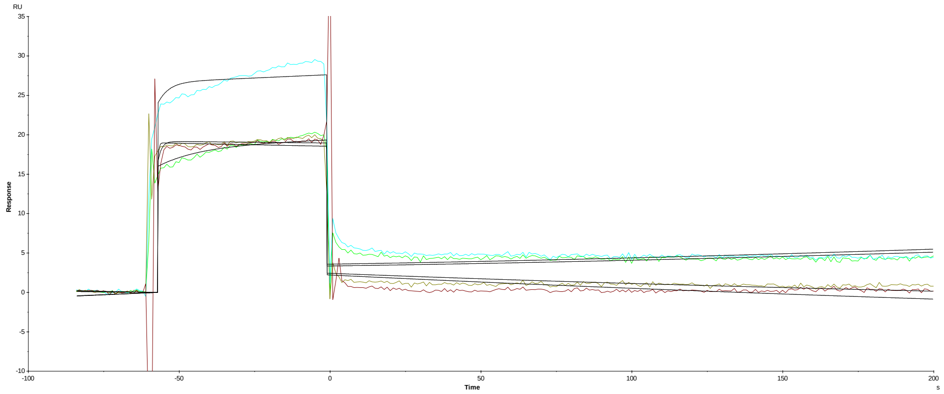
RU
35
30
25
20
15
10
5
0
-5
-10
-100
-50
0
50
100
150
200
s
Response
Time

5

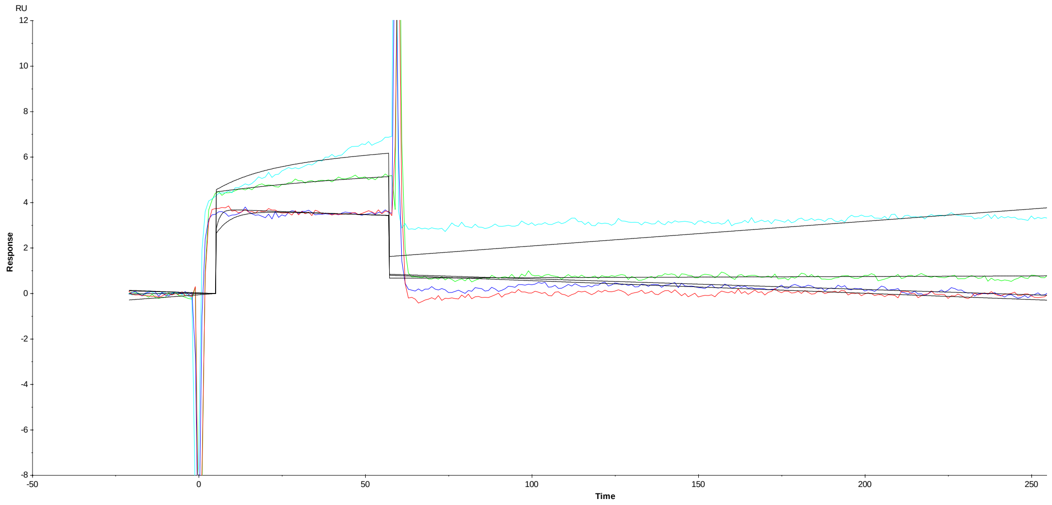
RU
12
10
8
6
4
2
0
-2
-4
-6
-8
-50
0
50
100
150
200
250
Response
Time

7

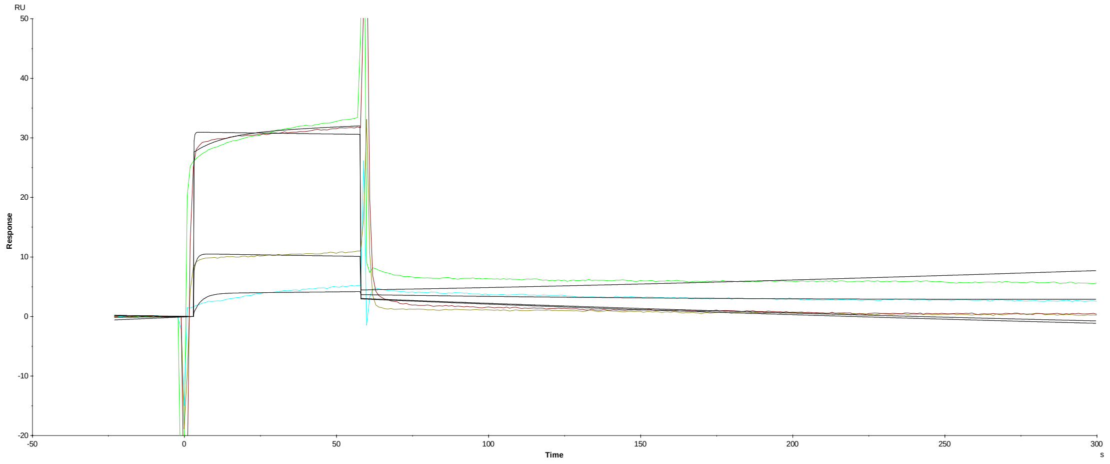

8

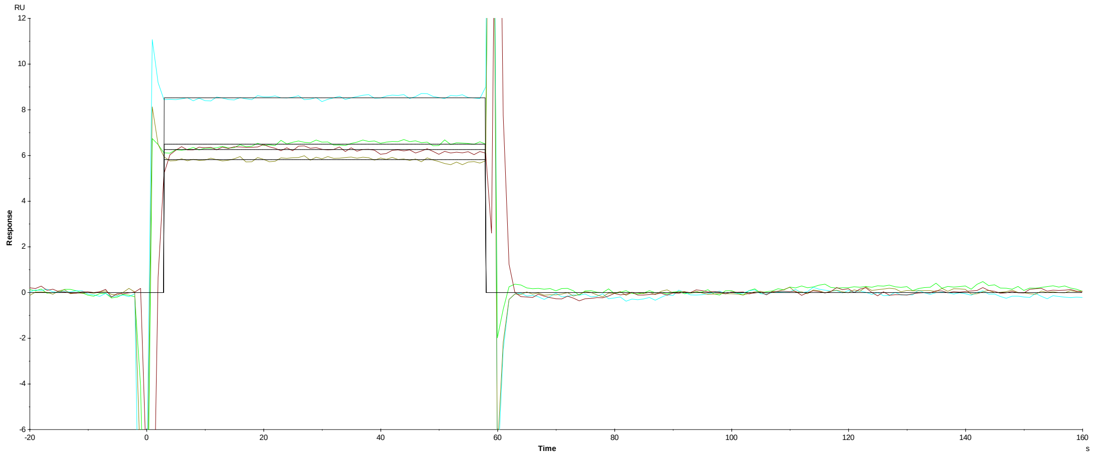

9

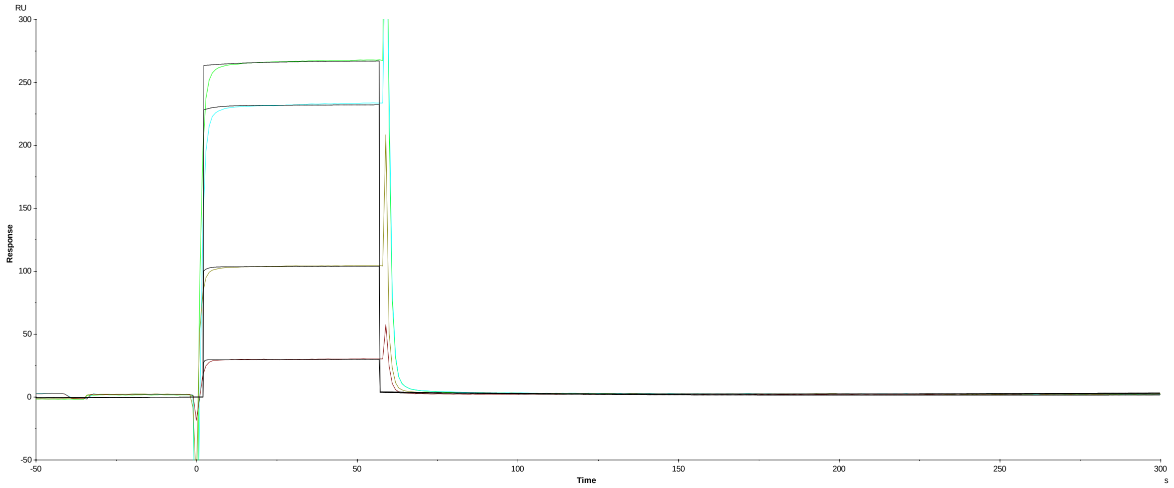

10

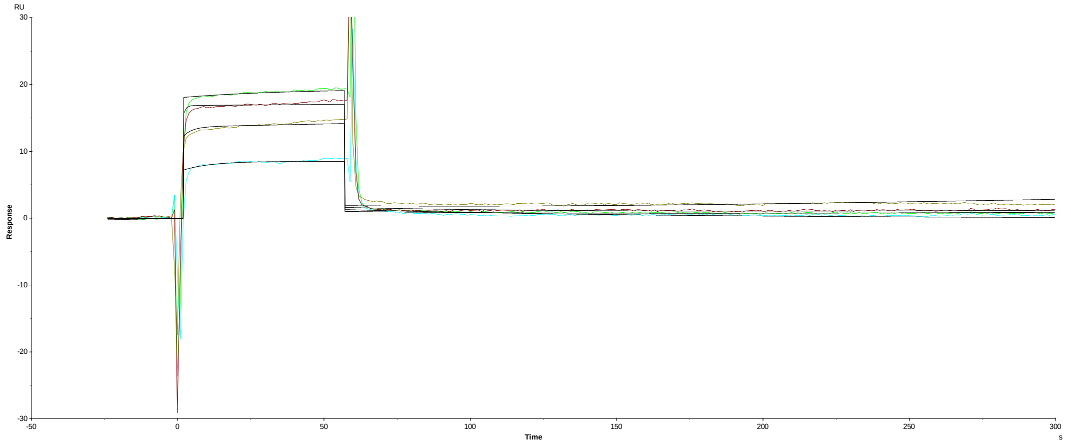
RU
30
20
10
0
-10
-20
-30
Response
-50
0
50
100
150
200
250
300
Time
s

11

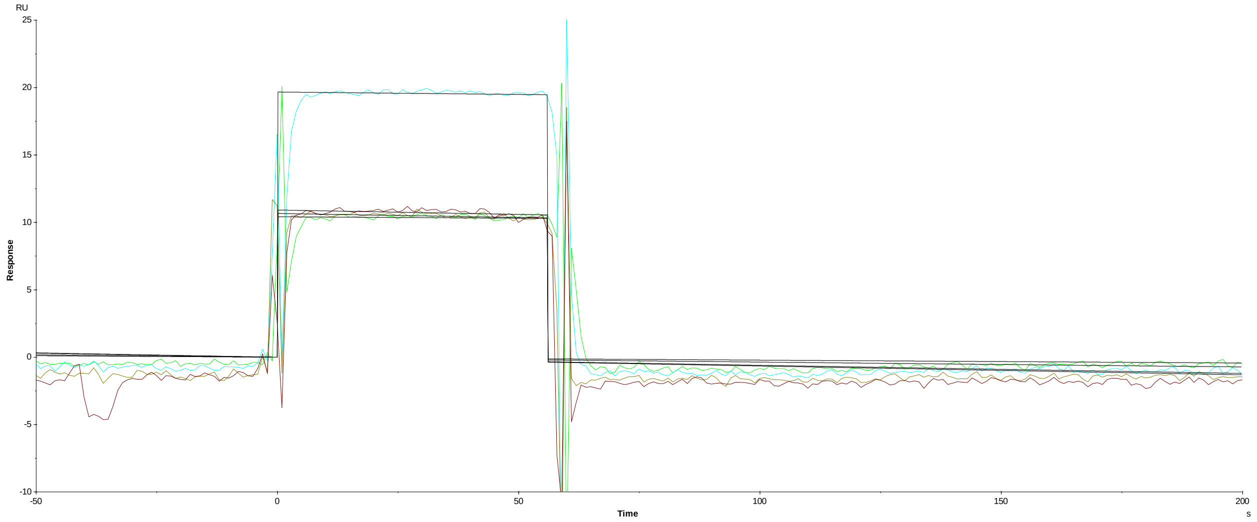
RU
25
20
15
10
5
0
-5
-10
Response
-50
0
50
100
150
200
Time
s

12

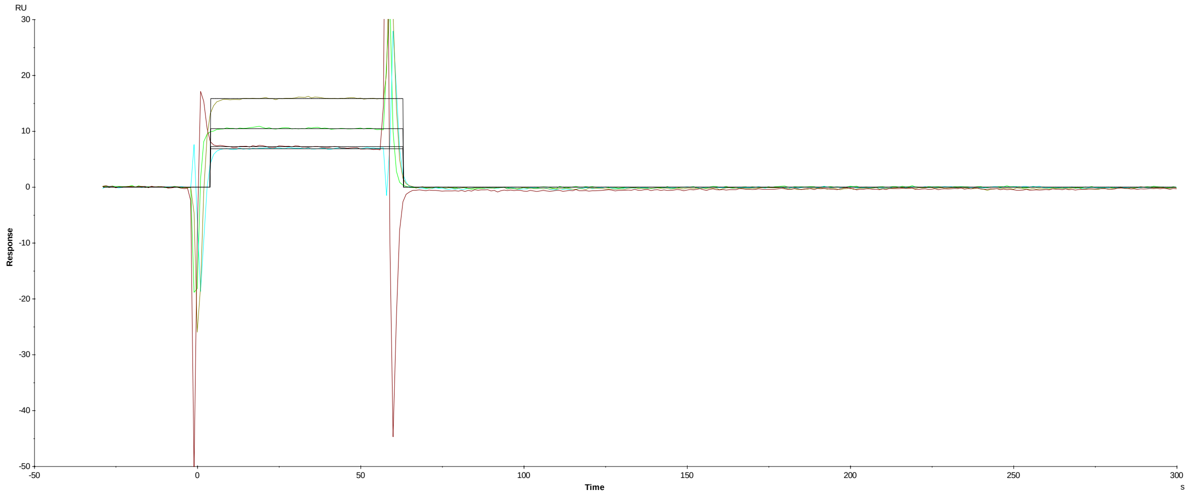
RU
30
20
10
0
-10
-20
-30
-40
-50
Response
-50
0
50
100
150
200
250
300
Time
s

13

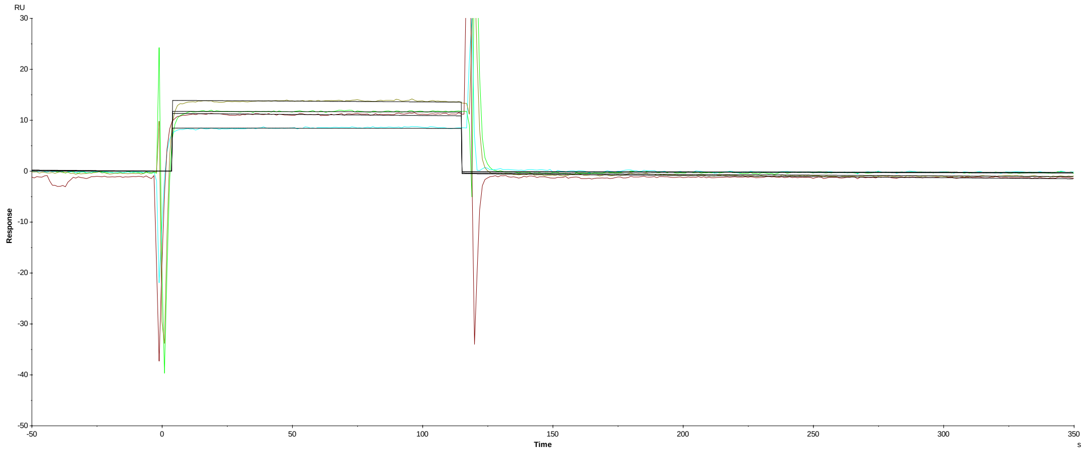
RU
30
20
10
0
-10
-20
-30
-40
-50
Response
-50
0
50
100
150
200
250
300
350
Time
s

14

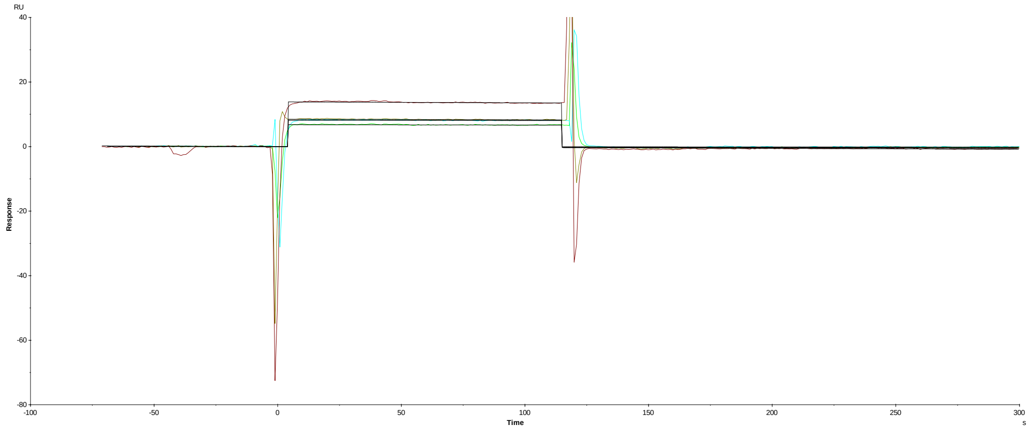
RU
40
20
0
-20
-40
-60
-80
Response
-100
-50
0
50
100
150
200
250
300
Time
s

15

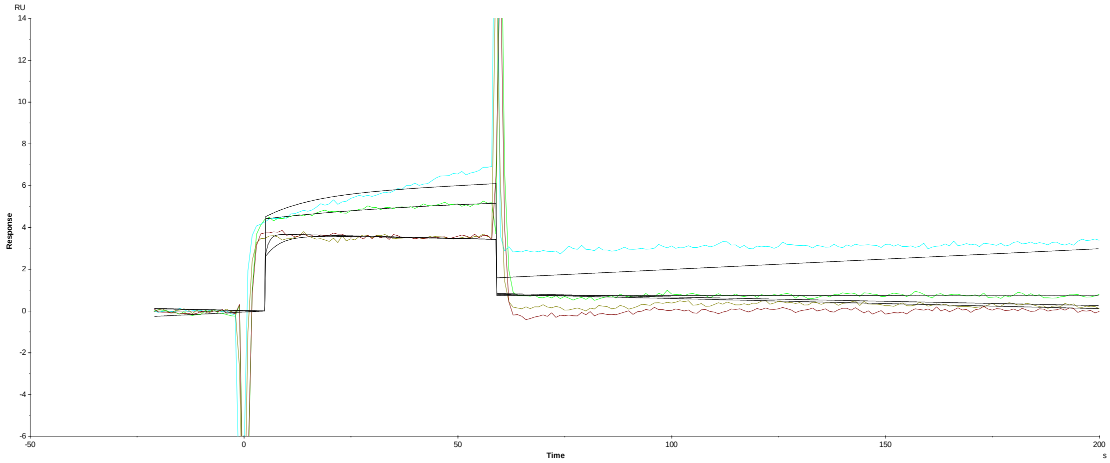
RU
14
12
10
8
6
4
2
0
-2
-4
-6
Response
-50
0
50
100
150
200
Time
s

20

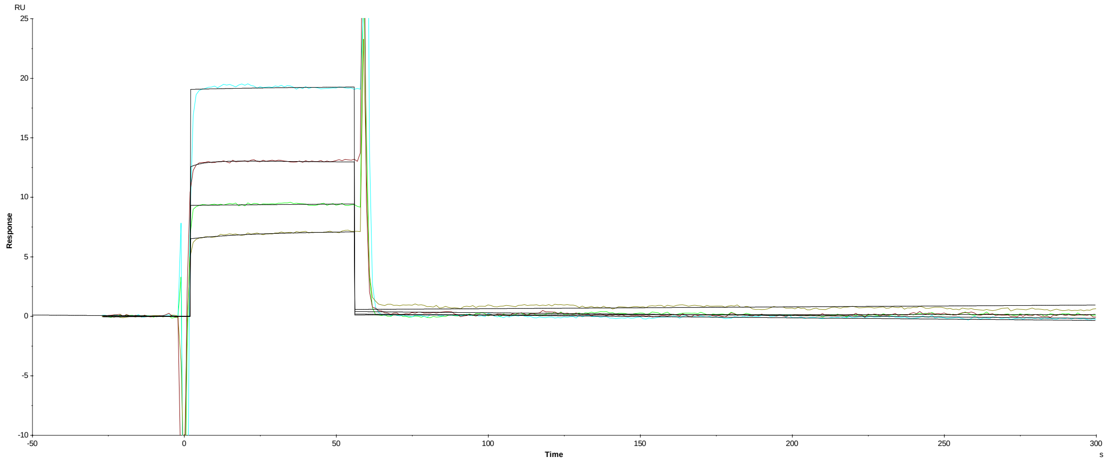
RU
25
20
15
10
5
0
-5
-10
Response
-50
0
50
100
150
200
250
300
Time
s

21

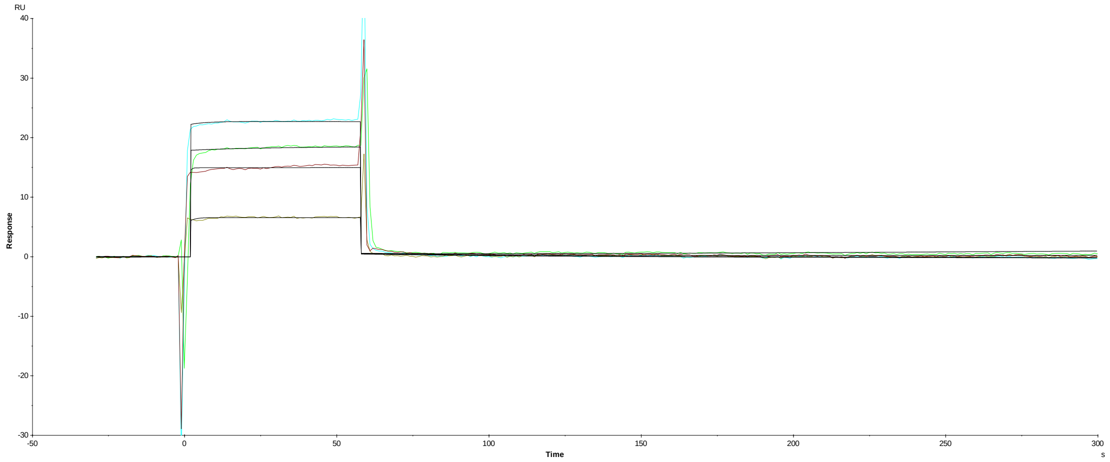
RU
40
30
20
10
0
-10
-20
-30
Response
-50
0
50
100
150
200
250
300
Time
s

22

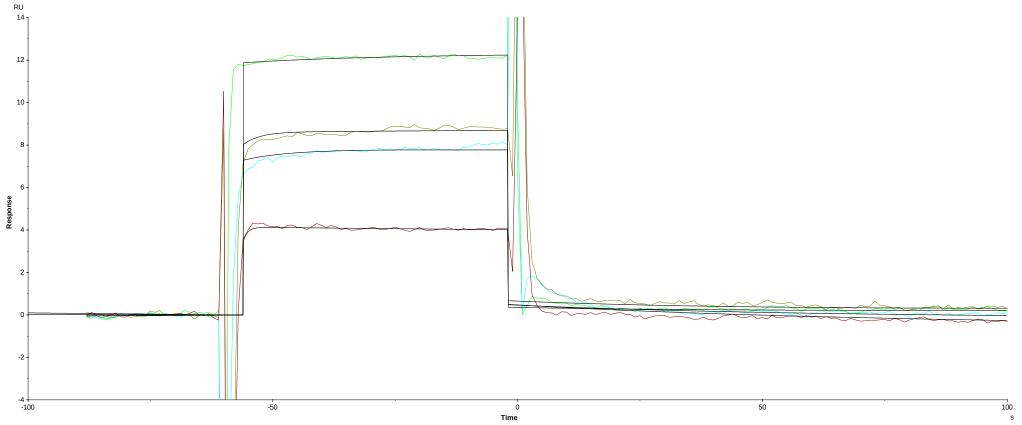
RU
14
12
10
8
6
4
2
0
-2
-4
Response
-100
-50
0
50
100
Time
s

26

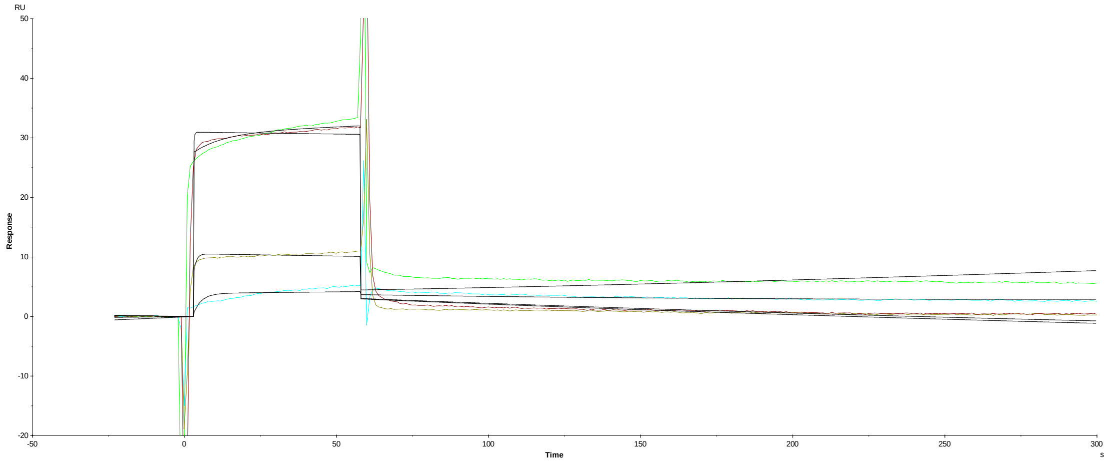

Supplementary Figure S7. SPR sensorgrams documenting the interaction between **1**, **4**, **5**, **7-15**, **20-22** and **26** Hsp90.

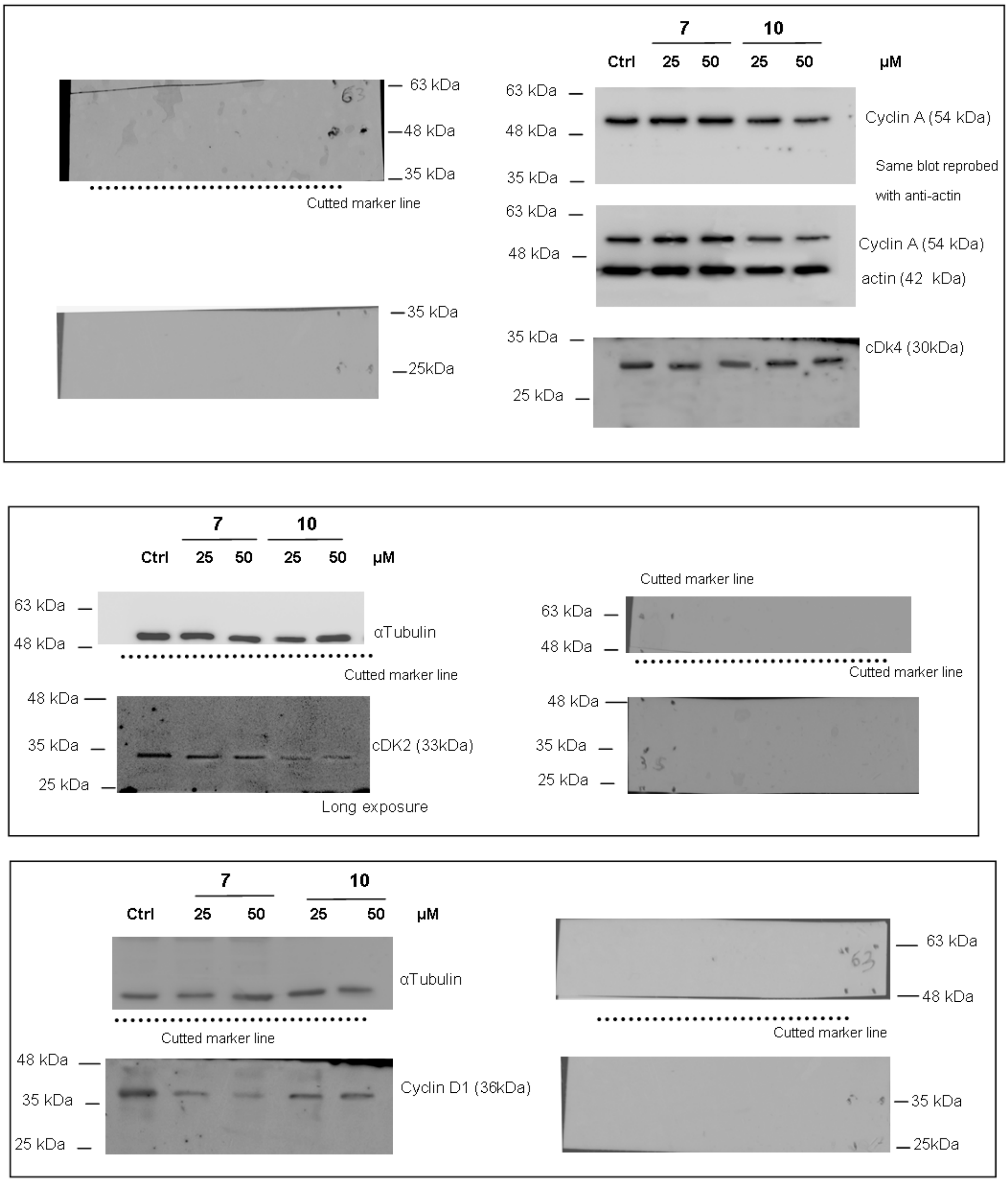

Supplementary Figure S8. Original images of western blots displayed in Figure 3A of the main text. In the figure was indicate the same filter cropped and probed with different primary antibody (Protein ladder :Opti-Protein XL Marker, Applied Biological Materials Inc., Canada).

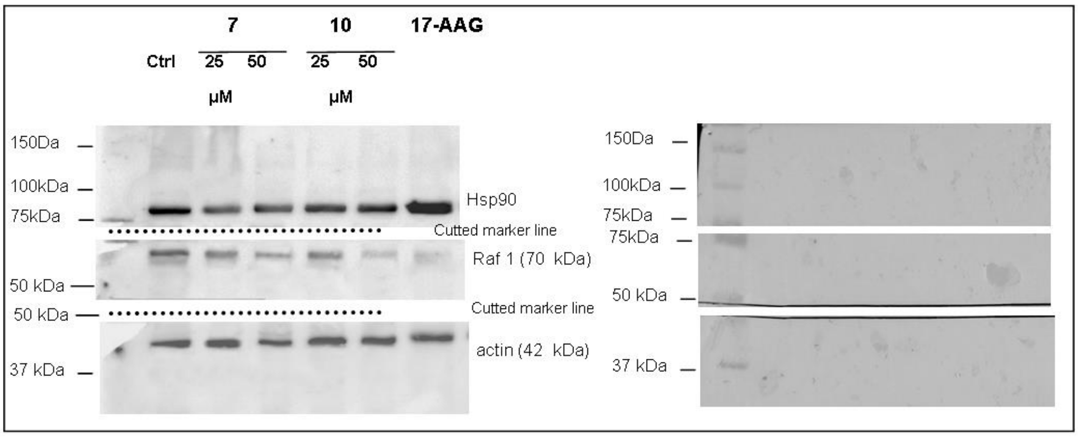
7
10
17-AAG
Ctrl
25
50
25
50
μM
μM
150Da
100kDa
75kDa
Hsp90
Cutted marker line
Raf 1 (70 kDa)
50 kDa
50 kDa
Cutted marker line
actin (42 kDa)
37 kDa
150Da
100kDa
75kDa
75kDa
50 kDa
37 kDa

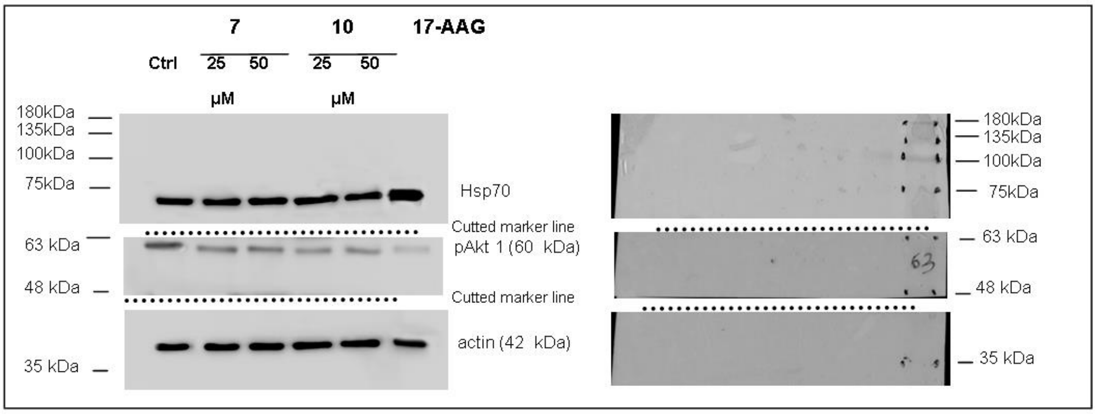
7
10
17-AAG
Ctrl
25
50
25
50
μM
μM
180kDa
135kDa
100kDa
75kDa
Hsp70
Cutted marker line
63 kDa
pAkt 1 (60 kDa)
48 kDa
Cutted marker line
actin (42 kDa)
35 kDa
180kDa
135kDa
100kDa
75kDa
63 kDa
63
48 kDa
35 kDa

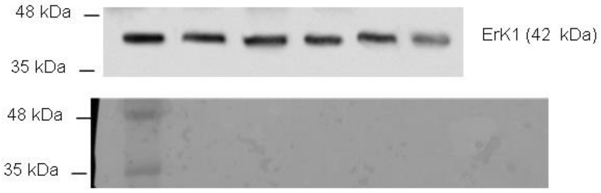
48 kDa
ErK1 (42 kDa)
35 kDa
48 kDa
35 kDa

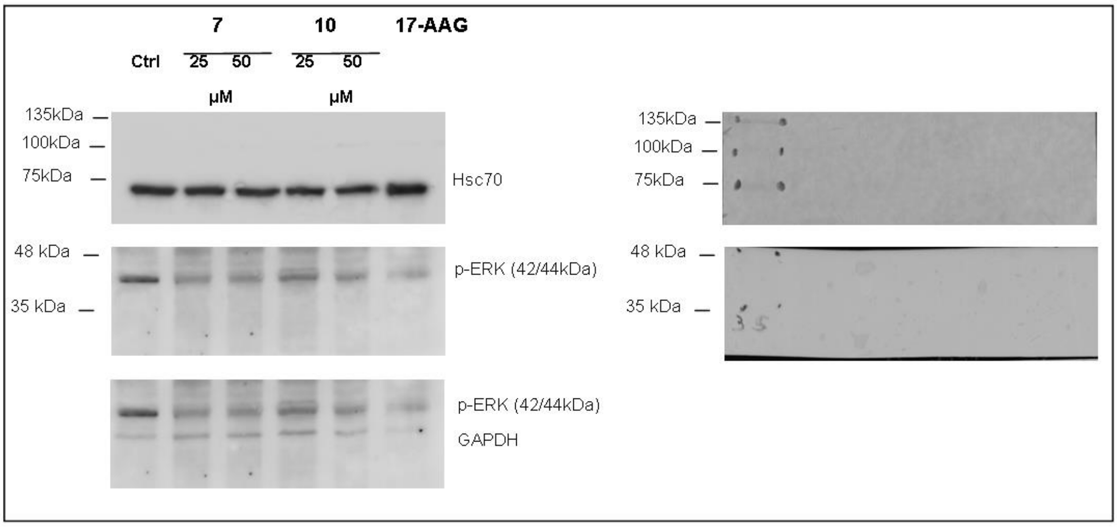

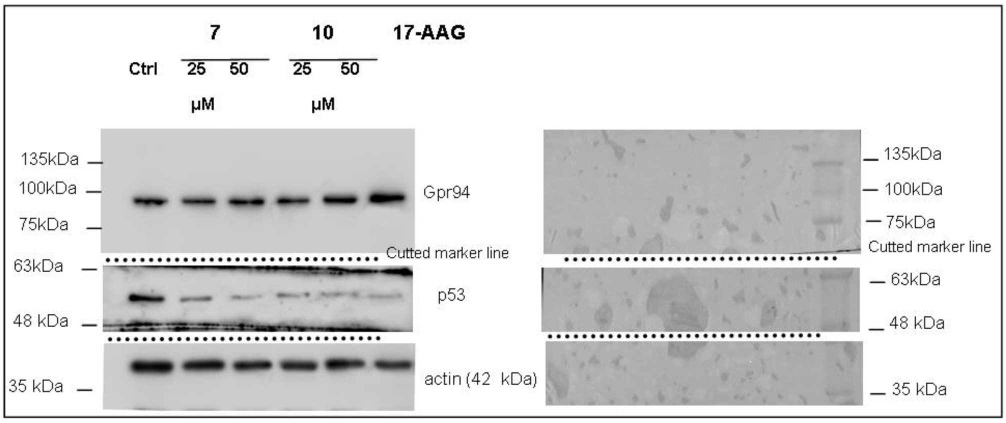

Supplementary Figure S9. Original images of western blots displayed in Figure 3B of the main text. The protein ladder was indicated (Opti-Protein XL Marker , Applied Biological Materials Inc., Canada; or Prestained Natural Protein Standard, Bio-Rad.). In the figure was indicate the same filter cropped and probed with different primary antibody.
